# Next-generation sequencing for pediatric-onset neuromuscular disorders unresolved by conventional diagnostic methods

**DOI:** 10.1038/s41390-025-04160-4

**Published:** 2025-06-10

**Authors:** Pimchanok Kulsirichawaroj, Mongkol Chanvanichtrakool, Pish Wattanadilokchatkun, Theeraphong Pho-iam, Chanin Limwongse, Surachai Likasitwattanakul, Kanokwan Boonyapisit, Oranee Sanmaneechai, Ichizo Nishino, Vorasuk Shotelersuk, Joohyun Han, Stephan Zuchner

**Affiliations:** 1 Division of Neurology, Department of Pediatrics, Faculty of Medicine, Siriraj Hospital, Mahidol University, Bangkok, Thailand; 2 Center of Research Excellence for Neuromuscular Diseases, Faculty of Medicine, Siriraj Hospital, Mahidol University, Bangkok, Thailand; 3 Pediatric Precision Medicine Center, Department of Pediatrics, Faculty of Medicine, Siriraj Hospital, Mahidol University, Bangkok, Thailand; 4 Siriraj Genomics, Office of the Dean, Faculty of Medicine, Siriraj Hospital, Mahidol University, Bangkok, Thailand; 5 Division of Medical Genetics, Department of Medicine, Faculty of Medicine, Siriraj Hospital, Mahidol University, Bangkok, Thailand; 6 Division of Neurology, Department of Medicine, Faculty of Medicine, Siriraj Hospital, Mahidol University, Bangkok, Thailand; 7 Department of Neuromuscular Research, National Institute of Neuroscience, National Center of Neurology and Psychiatry, Tokyo, Japan; 8 Center of Excellence for Medical Genomics, Department of Pediatrics, Faculty of Medicine, Chulalongkorn University, Bangkok, Thailand; 9 Excellence Center for Medical Genetics, King Chulalongkorn Memorial Hospital, the Thai Red Cross Society, Bangkok, Thailand; 10 Interdisciplinary Program of Medical Informatics, College of Medicine, Seoul National University, Seoul, Republic of Korea; 11 Department of Human Genetics and Hussman Institute for Human Genomics, University of Miami Miller School of Medicine, Miami, FL USA

## Abstract

**Background:**

Neuromuscular disorders (NMDs), rare diseases affecting the peripheral nervous system, often cause progressive weakness and systemic complications. Despite advances in genetic diagnostics, data from Southeast Asia remain limited. Ancestral variation may influence mutation spectra, revealing novel alleles and phenotypic diversity.

**Methods:**

We evaluated the diagnostic yield and clinical impact of targeted gene panel testing and exome sequencing in pediatric-onset NMDs at Siriraj Hospital, Thailand (2020‒2024). Patients with suspected genetic NMDs and negative single-gene tests underwent gene panel testing or exome sequencing. Genetic findings were classified as positive, probable, possible, or negative.

**Results:**

Among 135 patients, the overall diagnostic yield was 69.6% (94/135). Subgroup yields were 70.7% for inherited myopathies (53/75), 63.3% for inherited neuropathies (31/49), 90.0% for congenital myasthenic syndromes (9/10), and 100% for motor neuron diseases (1/1). Excluding patients with Duchenne muscular dystrophy, the diagnostic yield of inherited myopathies was 63.2% (36/57). Genetic diagnoses influenced clinical care in 87.2% of cases, prompting revised diagnoses, personalized treatments, enhanced surveillance, informed family planning, and accurate prognostication.

**Conclusions:**

NGS substantially enhances diagnostic accuracy and clinical management for pediatric NMDs. These findings support incorporating NGS into diagnostic workflows for suspected genetic NMDs, thereby optimizing patient care and advancing genetic insights.

**Impact:**

- Gene panel testing and exome sequencing demonstrate an approximately 70% diagnostic yield for pediatric neuromuscular disorders, exceeding yields reported in many other hereditary diseases.
- Genetic findings underscore potential differences in mutation spectra among Southeast Asian populations, which remain under-investigated relative to Western cohorts.
- Clinical implementation of next-generation sequencing confers substantial benefits, including more accurate diagnoses, personalized management, and informed family planning, ultimately improving care for pediatric neuromuscular disorders.

## Introduction

Neuromuscular disorders (NMDs) encompass a diverse group of peripheral nervous system conditions often marked by progressive weakness, numbness, contractures, and other systemic manifestations. They are rare diseases, with prevalence rates of 1‒10 per 100,000 worldwide, and they contribute significantly to pediatric morbidity.^1^ Diagnosing NMDs is challenging, given their phenotypic variability, overlapping clinical features, and the involvement of numerous genes.

Advancements in genetic testing, particularly next-generation sequencing (NGS), have transformed the diagnostic approach to NMDs. NGS technologies, including targeted gene panel (GP) testing and exome sequencing (ES), enable the simultaneous analysis of multiple genes, substantially improving diagnostic efficiency and accuracy.^2–4^ Additionally, NGS has facilitated the discovery of numerous disease genes, enhancing diagnostic precision and broadening our understanding of pathogenesis. It also guides patient management, facilitates genetic counseling, and paves the way for personalized medicine and targeted gene therapies for previously untreatable conditions.^5^

Studies have reported varying diagnostic yields for NGS approaches. For instance, targeted GP testing has proven effective in diagnosing certain neuromuscular conditions, whereas ES offers a broader analysis that may reveal novel genetic variants. The choice between targeted panels and ES often depends on clinical presentation and the suspected genetic etiology. However, there remains a gap in understanding the comparative effectiveness of these methods in pediatric-onset NMDs, particularly across different phenotypic subgroups.^4^ Given that genetic studies often focus on populations of European ancestry, there is a critical need to investigate the genomic landscape of Southeast Asian populations, such as Thai patients. This approach may uncover unique genetic variants and improve diagnostic yield for these underrepresented groups.

This study addressed this gap by evaluating the diagnostic yield of GP testing and ES in pediatric-onset genetic NMDs that remain undiagnosed despite conventional diagnostic tests. We had three objectives. First, we set out to determine the overall diagnostic yield of NGS in pediatric NMDs. Second, we aimed to analyze yields across subgroups (myopathies, neuropathies, congenital myasthenic syndromes, and motor neuron diseases). Third, we sought to assess the impact of genetic diagnoses on clinical care, including investigations, treatment, surveillance, family planning, and prognostication.

By providing robust evidence, this research can guide clinical practice, shape health policies, and advocate for the inclusion of advanced genetic testing technologies in health coverage packages. Ultimately, these efforts will improve patient outcomes and family care.

## Methods

### Ethics approval statement

The Siriraj Institutional Review Board approved the study (protocol number 343/2560 [EC4] and approval number Si-501/2017), and the research was conducted in accordance with the principles of the Declaration of Helsinki. Informed consent to collect 3–5 mL of peripheral blood was obtained from each participant and their parent(s). Written informed consent for publication was also obtained.

### Study design and patient population

This investigation was a combined prospective and retrospective cohort study of patients suspected of having pediatric-onset genetic NMDs (age <21 years). These patients were referred to the Pediatric Neuromuscular Clinic at Siriraj Hospital, Thailand, between December 2020 and October 2024. Each enrolled patient was evaluated by a pediatric neurologist and a neuromuscular specialist at one or more clinic visits.

We collected comprehensive data, including sex, age at onset, and age at the first neurology visit. We also recorded disease presentation, family history, physical examination findings, and age at both genetic testing and test reporting. Additional data included results from conventional investigations such as blood assays, imaging, muscle biopsy, nerve electrophysiological studies, single-gene testing, and other genetic tests.

In patients with neuropathy, nerve electrophysiological studies classified cases as demyelinating, axonal, or intermediate, based on compound muscle action potential (CMAP), sensory nerve action potential (SNAP), and conduction velocity (CV). A CV <35 m/s in the upper extremity indicated demyelination, 35‒45 m/s was classified as intermediate, and >45 m/s was deemed as axonal. Absent or indiscernible CMAP and SNAP responses were categorized as undetermined.

“Time to first neurology visit” was defined as the duration from symptom onset to the first neurology appointment. “Time to first NGS testing” was the duration from symptom onset to the first NGS test. “Time to NGS result” was the duration from symptom onset to the reporting of NGS results. Supplementary Table S1 lists the single-gene and other genetic tests that a neurologist might order individually based on the provisional diagnosis.

After evaluation, patients were grouped into four subcategories: inherited myopathy, inherited neuropathy, congenital myasthenic syndrome, and motor neuron disease. The inherited myopathy subgroup was further divided into congenital myopathy, Duchenne muscular dystrophy with negative multiplex ligation-dependent probe amplification, other muscular dystrophies, metabolic/mitochondrial myopathies, and muscle channelopathies.

All patients underwent at least one form of genetic testing (either a GP test or ES). The exclusion criteria were incomplete medical records, a clinical diagnosis of facioscapulohumeral muscular dystrophy, or a diagnosis confirmed solely by single-gene testing or targeted variant sequencing.

### NGS

All recruited patients underwent NGS, using targeted GP or ES, depending on clinical indication and test availability. Genomic DNA was extracted from leukocytes using a Gentra Puregene Blood Kit (Qiagen, Hilden, Germany). Alternatively, genomic DNA was prepared as an Illumina sequencing library enriched by the SureSelect Human All Exon V5 Kit (Agilent, Santa Clara, CA).

Targeted GP testing was conducted at the National Center of Neurology and Psychiatry in Japan or at INVITAE Corporation in the United States. These panels were selected according to each patient’s clinical phenotype. They covered a broad spectrum of disorders, including NMDs, muscular dystrophy, congenital myopathy, malignant hyperthermia, periodic paralysis, neuropathy, Charcot‒Marie‒Tooth disease, and congenital myasthenic syndrome.

Variant calling, ES, filtering, and pathogenicity analyses were performed at multiple institutions. These included Sungkyunkwan University School of Medicine and 3billion Co Ltd in Korea and the University of South Florida in the United States. Additional analyses were conducted at the Faculties of Medicine at Chulalongkorn University and Siriraj Hospital in Bangkok, Thailand. All candidate variants underwent assessments by clinical geneticists and neurologists. They classified these variants according to the American College of Medical Genetics and Genomics/Association for Molecular Pathology criteria.^6^

Segregation analysis of variants in parents was performed to evaluate cis/trans phase in autosomal recessive (AR) inheritance. It also helped determine de novo origin or inheritance in autosomal dominant cases and identified patients with multiple potential pathogenic variants, variants of uncertain significance, or novel variants.

Genetic testing results were classified into four categories: positive, probable, possible, and negative. A result was considered “positive” if pathogenic or likely pathogenic (P/LP) variants explained the phenotype and followed the expected inheritance pattern. “Probable” results included variants strongly associated with the phenotype but lacking definitive evidence. Examples included two P/LP variants in AR genes with unconfirmed phasing. They also encompassed one P/LP variant in an AR gene or an autosomal dominant gene with unknown parental phenotype or genotype. “Possible” results involved variants of uncertain significance in genes potentially linked to the phenotype but lacking sufficient evidence for pathogenicity, including one or two variants of uncertain significance in AR genes. “Negative” results indicated no P/LP variants relevant to the phenotype. Disease-causing variants were defined as positive or probable results.

Cases were subsequently classified as solved (positive or probable genetic findings) or unsolved (possible or negative results). The diagnostic yield was calculated as the proportion of patients with a disease-causing variant (solved cases) identified by GP testing or ES, divided by the total number of patients tested.^7^ Some variants reported here have been published previously (PMID: 32978031, 36697461, 37927275).^8–10^

For solved cases, further analysis focused on changes in diagnosis and management, where genetic test results directly influenced the patient’s care plan. This included adjustments to diagnostic investigations, such as avoiding invasive procedures based on the genetic diagnosis and tailoring treatments to target the underlying genetic condition. Specialized surveillance strategies were also implemented to monitor disease progression or associated complications, while family counseling addressed inheritance risks, reproductive options, and prognostic information regarding the expected disease course.

Unsolved cases were further investigated through either retesting (using either ES or GP testing) or short-read genome sequencing conducted under a separate project. The selection between retesting and genome sequencing depended on test availability and research funding at the time. The results from the retesting were incorporated into the overall diagnostic yield calculation and were assessed for their clinical impact.

## Results

### Diagnostic yield

**Fig. 1:**
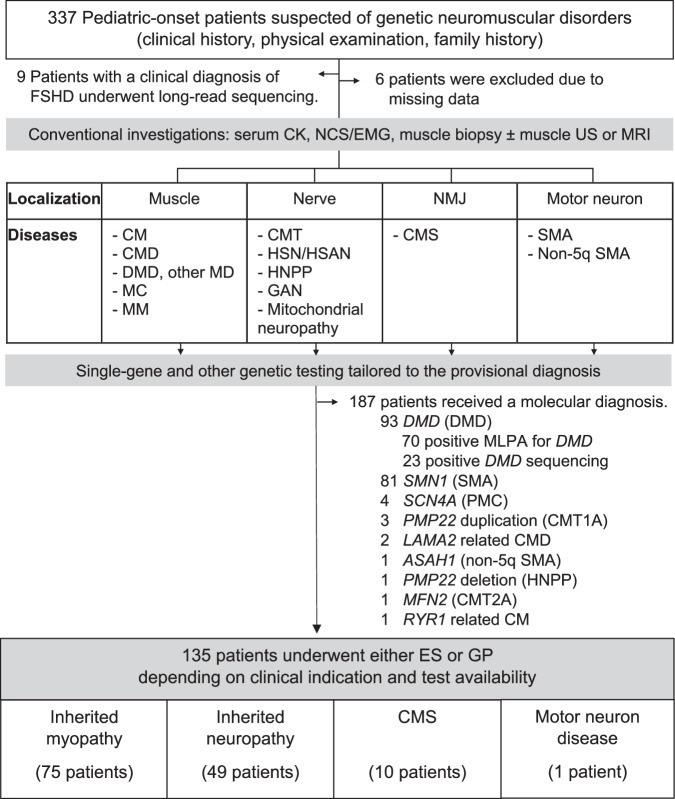
Patient enrollment workflow for genetic neuromuscular disorder diagnosis. CK creatine kinase, CM congenital myopathy, CMD congenital muscular dystrophy, CMS congenital myasthenic syndrome, CMT Charcot–Marie–Tooth, DMD Duchenne muscular dystrophy, ES exome sequencing, FSHD facioscapulohumeral muscular dystrophy, GAN giant axonal neuropathy, GP gene panel, HNPP hereditary neuropathy with liability to pressure palsy, HSN/HSAN hereditary sensory neuropathy/hereditary sensory and autonomic neuropathy, MC muscle channelopathy, MD muscular dystrophy, MM metabolic/mitochondrial myopathy, MRI magnetic resonance imaging, NCS/EMG, nerve conduction study/electromyography, PMC paramyotonia congenita, NMJ neuromuscular junction, SMA spinal muscular atrophy, US, ultrasound.

A total of 337 patients suspected of having genetic NMDs were initially assessed based on clinical history, physical examination, and family history (Fig. 1). Six patients were excluded due to missing data, nine had a clinical diagnosis of facioscapulohumeral muscular dystrophy, and 187 received a molecular diagnosis through single-gene or target variant testing. The remaining 135 patients, from 130 unrelated families and without a confirmed molecular diagnosis, underwent GP testing and/or ES. These individuals were subsequently classified as having inherited myopathy (*n* = 75), inherited neuropathy (*n* = 49), congenital myasthenic syndrome (*n* = 10), or motor neuron disease (*n* = 1).

Table 1 summarizes the demographic data for the 135 patients who underwent NGS. Overall, 82 (60.7%) were male. The median age at onset was 2.0 years (interquartile range [IQR], 0.5‒4.0). The median time to first neurology visit was 2.8 years (IQR, 1.3‒4.7), the median time to first NGS testing was 4.8 years (IQR, 2.2‒9.2), and the median time to NGS result was 5.7 years (IQR, 2.5‒10.7). A family history of NMDs was reported in 31 (23.0%) patients, and consanguinity was present in 7 (5.2%).

**Table 1:** Demographic and clinical characteristics of pediatric-onset neuromuscular patients undergoing next-generation sequencing.

Characteristics	Overall ^d^ *N* = 135	Inherited myopathy *N* = 75	Inherited neuropathy *N* = 49	CMS *N* = 10
Sex, male	82 (60.7%)	51 (68.0%)	25 (51.0%)	6 (60.0%)
Age at onset (years)	2.0 (0.5, 4.0)	1.6 (0.4, 4.0)	2.0 (1.0, 6.0)	0.3 (0.0, 4.0)
Age at first neurology visit (years)	6.2 (2.8, 10.3)	5.3 (2.4, 8.5)	9.3 (4.7, 12.7)	2.6 (1.5, 16.1)
Age at first NGS test (years)	8.4 (5.0, 13.0)	7.6 (4.9, 11.5)	12.3 (7.0, 16.7)	5.2 (1.9, 19.4)
Time to first neurology visit ^a^ (years)	2.8 (1.3, 4.7)	2.1 (0.8, 4.0)	3.7 (2.5, 6.1)	2.6 (1.0, 9.9)
Time to first NGS test ^b^ (years)	4.8 (2.2, 9.2)	4.1 (1.4, 7.2)	7.1 (3.3, 13.6)	4.7 (1.9, 15.4)
Time to NGS result ^c^ (years)	5.7 (2.5, 10.7)	4.5 (1.7, 8.7)	8.8 (4.8, 14.3)	5.2 (2.8, 17.3)
Family history	31 (23.0%)	17 (22.7%)	10 (20.4%)	3 (30.0%)
Consanguinity	7 (5.2%)	4 (5.3%)	2 (4.1%)	1 (10.0%)

Data are displayed as either median (interquartile range) or *n* (%).

*CMS* congenital myasthenic syndrome, *NGS* next-generation sequencing.

^a^“Time to first neurology visit” is defined as the duration from symptom onset to the first neurology visit.

^b^“Time to first NGS test” is defined as the duration from symptom onset to the first NGS test.

^c“^Time to NGS result” is defined as the duration between symptom onset and the reporting of NGS results.

^d^Data also includes motor neuron disease (*n* = 1).

**Fig. 2:**
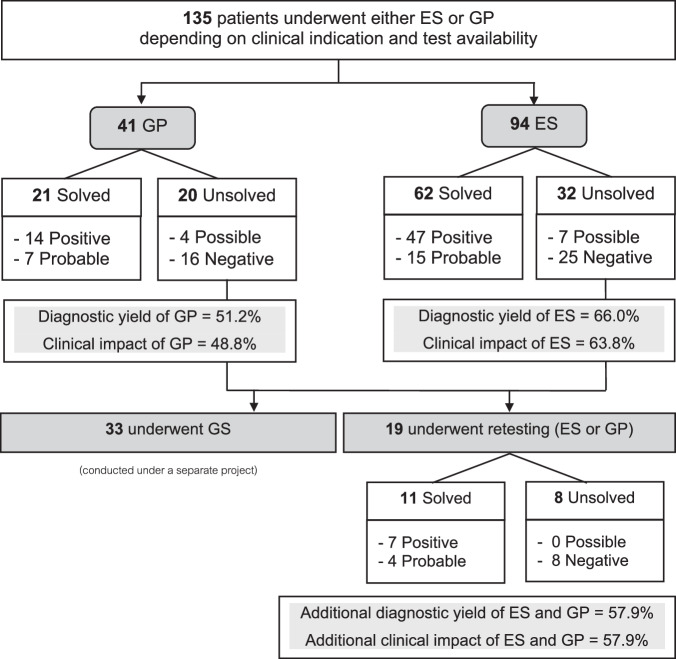
Diagnostic outcomes and test sequences for NGS in patients suspected genetic neuromuscular disorders. ES exome sequencing, GP gene panel, GS genome sequencing (short-read), NGS next-generation sequencing.

Across these 135 patients, 44 targeted GP tests, and 112 ES tests were performed. Most (*n* = 116) underwent only one NGS test, whereas 19 underwent retesting (Fig. 2). Among the ES cases, 72 were singleton, 2 were duo, and 38 were trio analyses.

**Fig. 3:**
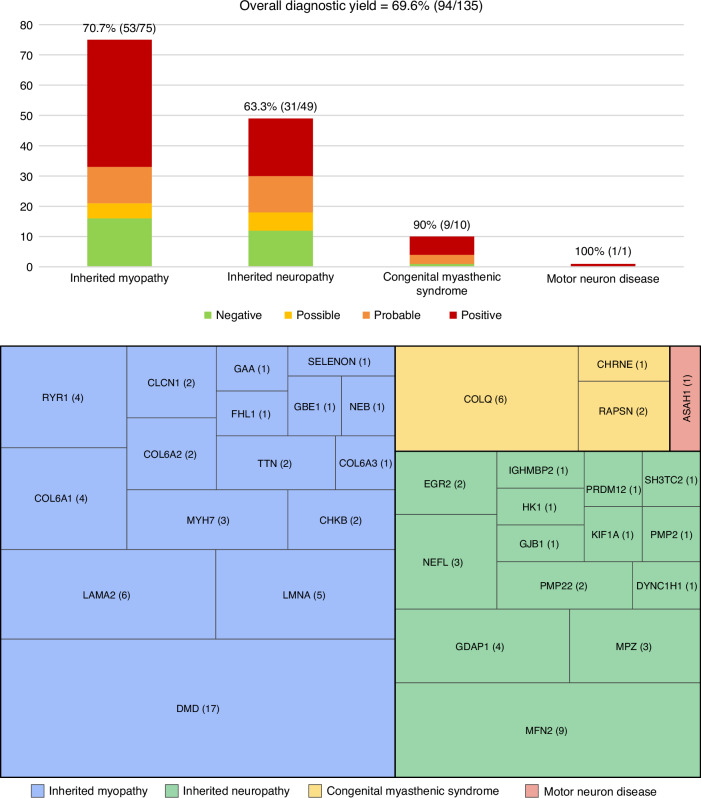
Diagnostic yield of next-generation sequencing in pediatric-onset neuromuscular disorders and distribution of causative genes. The diagnostic yield is the ratio of positive and probable cases to all cases evaluated.

Based on final genetic results, 68 cases were positive, 26 were probable, 11 were possible, and 30 were negative. Thus, the overall diagnostic yield was 69.6% (94/135; Fig. 3). In total, 104 pathogenic/likely pathogenic variants were identified in 34 genes, along with 9 variants of uncertain significance. The clinical characteristics of these patients, the disease-associated genes, and the molecular findings are detailed in Supplementary Tables S2 and S3.

#### Inherited myopathy

The overall diagnostic yield for inherited myopathies was 70.7% (53/75). When patients with Duchenne muscular dystrophy (DMD) were excluded, the diagnostic yield was 63.2% (36/57). In the congenital myopathy subgroup, 12 of 22 patients (54.5%) had disease-causing variants in *RYR1* (*n *= 4), *MYH7* (*n *= 3), *TTN* (*n *= 2), *FHL1* (*n *= 1), *NEB* (*n *= 1), and *SELENON* (*n *= 1).

For muscular dystrophy, NGS detected the responsible variants in 17 of 18 multiplex ligation-dependent probe amplification-negative Duchenne muscular dystrophy patients, comprising 16 point mutations (8 frameshifts, 3 splice-site, 3 nonsense, and 2 in-frame deletions). Two unrelated patients shared the c.10108C>T (p.Arg3370*) variant. Among other muscular dystrophies, 20 of 24 patients (83.3%) had variants in *LAMA2* (*n *= 6), *LMNA* (*n *= 5), *COL6A1* (*n *= 4), *CHKB* (*n *= 2), *COL6A2* (*n *= 2), and *COL6A3* (*n *= 1).

In metabolic or mitochondrial myopathy, 2 of 8 patients (25%) carried variants in *GAA* (*n *= 1) and *GBE1* (*n *= 1). The patient with *GAA* variants, diagnosed with late-onset glycogen storage disease type 2, was eligible for Myozyme. The patient with *GBE1* variants, who had severe glycogen storage disease type 4, experienced respiratory failure from birth and required full ventilatory support. In muscle channelopathy, *CLCN1* variants were identified in 2 of 3 patients presenting with typical myotonia and muscle hypertrophy.

#### Inherited neuropathy

The diagnostic yield for inherited neuropathy was 63.3% (31/49). Among 44 patients with Charcot‒Marie‒Tooth disease, 65.9% (29/44) had P/LP variants: 11 of 24 in the axonal type, 11 of 12 in the demyelinating type, 2 of 2 in the intermediate type, and 5 of 6 in undetermined types. These variants were identified in *MFN2* (*n *= 9), *GDAP1* (*n *= 4), *MPZ* (*n *= 3), *NEFL* (*n *= 3), *EGR2* (*n *= 2), *PMP22* (*n *= 2), *DYNC1H1* (*n *= 1), *GJB1* (*n *= 1), *HK1* (*n *= 1), *IGHMBP2* (*n *= 1), *PMP2* (*n *= 1), and *SH3TC2* (*n *= 1). Notably, the same variants appeared in unrelated patients: *GDAP1* c.368A>G, *MFN2* c.1090C>T, and *NEFL* c.280C>T (Supplementary Table S3). Among five patients with hereditary sensory neuropathy, 2 of 5 (40%) had pathogenic variants: one in *PRDM12* and one in *KIF1A*. The *PRDM12* variant was associated with early-onset symptoms, including constipation, corneal ulcers, self-mutilation (lip and finger autoamputation), and axonal sensory polyneuropathy.

#### Congenital myasthenic syndrome

NGS identified disease-causing variants in 9 of 10 congenital myasthenic syndrome patients (90.0%). Most mutations were found in *COLQ* (*n *= 6), followed by *RAPSN* (*n *= 2) and *CHRNE* (*n *= 1). The age at onset among *COLQ* carriers ranged from birth to 10 years. Notably, all three patients with infantile-onset *COLQ* mutations experienced recurrent respiratory failure prior to developing obvious ptosis and ophthalmoplegia.

#### Motor neuron disease

A disease-causing variant was identified in one patient with suspected spinal muscular atrophy. This two-year-old girl presented with delayed motor milestones and tremors beginning at 1.5 years. Clinical examination revealed a myopathic face, hypotonia, proximal weakness, and areflexia. Creatine kinase was 1475 U/L, brain magnetic resonance imaging was normal, and muscle biopsy showed neurogenic changes. Multiplex ligation-dependent probe amplification for *SMN1* was negative. Trio-ES identified compound heterozygous *ASAH1* variants (c.1A>T and c.770T>C), confirming spinal muscular atrophy with progressive myoclonic epilepsy. By 3 years, she had developed respiratory failure due to bilateral diaphragmatic paralysis and drug-resistant epilepsy requiring three antiseizure medications.

### Impact on diagnosis and clinical management

**Fig. 4:**
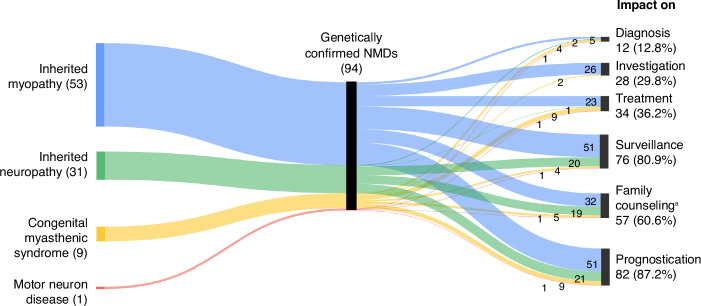
Impact of next-generation sequencing on clinical diagnosis and management in 94 patients with genetically confirmed neuromuscular disorders (NMDs). ^a^100% of diagnosed patients were able to receive accurate genetic counselling and genetic testing options for family members.

A genetic diagnosis via ES and GP testing improved patient care in 87.2% of cases by offering six key benefits (Fig. 4). It led to revised diagnoses in 12 patients, primarily in the congenital myasthenic syndrome group, and prevented unnecessary investigations in 26 cases, including muscle biopsies. Genetic findings enabled targeted therapies such as enzyme replacement (Myozyme) for glycogen storage disease type 2 and read-through therapy (Ataluren) for nonsense Duchenne muscular dystrophy variants. Steroids plus angiotensin-converting enzyme inhibitors or angiotensin receptor blockers were also used in Duchenne muscular dystrophy. Salbutamol and ephedrine were indicated for *COLQ*-related congenital myasthenic syndrome, and harmful medications (e.g., acetylcholinesterase inhibitors) were avoided. Misdiagnosed patients discontinued immunosuppressants. Genetic results also improved surveillance and management of comorbidities, facilitated family planning through preimplantation and prenatal testing, and enhanced prognostic accuracy. These findings emphasize the transformative role of ES and GP testing in precision medicine (Supplementary Table S2).

## Discussion

In this study, we employed NGS using targeted GP testing and/or ES to evaluate 135 patients from 130 families. These patients remained undiagnosed after standard investigations for suspected genetic NMDs. Our overall diagnostic yield was 69.6%, consistent with previous studies that reported yields between 26% and 78% for ES and GP tests in pediatric NMDs.^11–15^ These findings underscore the power of NGS in resolving genetically heterogeneous conditions.

The high diagnostic rate of 69.6% can be attributed to several factors. First, patients referred to our tertiary care centers were highly selected and had a strong suspicion of genetic disorders. Most presented with infantile-onset weakness. Second, the inclusion of multiplex ligation-dependent probe amplification-negative Duchenne muscular dystrophy patients and congenital myasthenic syndrome patients with abnormal repetitive nerve stimulation results increased our positive rate. Both conditions are associated with high diagnostic yields. Some individuals who were diagnosed with inherited muscle diseases before the genetic era had already undergone muscle biopsy, which helped guide the search for causative genes. Finally, the use of NGS significantly contributed to the overall diagnostic yield.

The diagnostic yield varied across NMDs, with inherited myopathies showing the highest yield (70.7%). Among muscular dystrophies, NGS identified the genetic cause in 94.4% (17/18) of multiplex ligation-dependent probe amplification-negative Duchenne muscular dystrophy patients, aligning with previous reports that demonstrate detection rates of 90‒94% in similar cases.^16,17^ This highlights the utility of NGS in detecting point mutations and small deletions that conventional copy number assays often miss. In other muscular dystrophies and congenital myopathies, *LAMA2*-, *COL6*-, and *RYR1*-related conditions were most frequently implicated, consistent with prior findings on early-onset muscle diseases.^9,11,18–20^ Notably, two unrelated patients shared the same c.598del (p.Gln200Argfs*11) homozygous *CHKB* mutation, presenting with delayed walking, proximal muscle weakness, and elevated CK levels (1279–1545 U/L), as described in a similar Chinese case.^21^ This may suggest a founder effect in the Asian population, warranting further investigation. In inherited myopathies, ES is especially valuable for patients whose muscle biopsy results are nonspecific. Conversely, where ES reveals variants of uncertain significance, muscle biopsy findings can support a definitive diagnosis, underscoring the complementary roles of these diagnostic tools.

P/LP variants were identified in 63.3% of patients with inherited neuropathy, in line with previous reports on pediatric inherited neuropathy, which show diagnostic yields ranging from 66.7% to 80.6%.^10,22–26^ Excluding the *PMP22* duplication, *MFN2* and *GDAP1* are most frequently implicated in the axonal type, whereas *MPZ* and *NEFL* predominate in the demyelinating type of early-onset Charcot‒Marie‒Tooth disease. These findings mirror those reported for the Han Chinese population in Taiwan.^25^

In a subset of four patients (Patient IDs: CM5, CM7, MD12, and CMS9), a single LP/P variant in an autosomal recessive gene was classified as a “probable” diagnosis based on strong phenotypic concordance, supported by clinical, electrophysiological, or histopathological findings. Although these cases do not meet the criteria for a confirmed molecular diagnosis, they likely represent instances where a second pathogenic variant was not detected due to technical limitations of ES or GP testing (e.g., deep intronic, structural, or regulatory region variants). Potential next steps to resolve these cases include whole genome sequencing, RNA sequencing, or periodic reanalysis as genomic databases continue to evolve.

The relatively low number of variants of uncertain significance (VUS) rate in our cohort likely reflects selective reporting and a targeted, phenotype-driven testing approach. VUS were reported only when clinically relevant or when follow-up testing was feasible, while unrelated variants were excluded. Additionally, the limited access to broad ES/GP—typically reserved for cases with high clinical suspicion—likely reduced the detection of incidental or less interpretable variants, contributing to a lower VUS rate compared to studies with routine ES/GP access.^27^

To highlight the importance of population-specific data, we reviewed the allele frequencies of variants identified in our Thai cohort (Supplementary Table S3) and found that several were either absent or extremely rare in large databases (e.g., gnomAD), underscoring the need to expand genetic studies in underrepresented populations.

Regarding gene distribution, we found that the patterns in the inherited myopathy and inherited neuropathy groups were similar to those reported in other ethnic populations. However, in the congenital myasthenic syndrome (CMS) group, *COLQ* was the most frequently affected gene—an observation that contrasts with previous reports, in which *COLQ* accounts for approximately 10–15% of CMS cases.^28–30^ Notably, five patients in our cohort harbored the splice-site variant c.393+1G>A, which has been previously reported in Chinese siblings.^30–32^ While this finding may suggest a unique genetic profile within our Asian population, we acknowledge the limitation posed by the small sample size in the CMS group. We recommend that future studies involving larger, more regionally representative cohorts be conducted to validate these findings.

NGS testing influenced clinical management in 87.2% of diagnosed cases by facilitating diagnostic revisions, altering investigative approaches, guiding genetic counseling, and informing reproductive decisions.^15,33,34^ It also enabled personalized care strategies, including disease surveillance, comorbidity management, prognostication, and family planning. Although all participants received genetic counseling, a significant impact on family counseling occurred in 60% of cases, where the diagnosis prompted actionable steps for relatives. The relatively low percentage of diagnosis changes may be due to our study’s setting in a pediatric center specializing in NMDs. Patients at this center receive expert evaluation from pediatric neurologists and a neuromuscular specialist. Even in the absence of specific treatments or clinical trials, a genetic diagnosis provides substantial benefits, such as shortening the diagnostic journey, clarifying disease etiology, and promoting education, empowerment, and psychosocial support. Family members may also undergo screening, presymptomatic testing, and potentially presymptomatic treatment.^35^

Increasing evidence demonstrates that NGS technology, including GP and ES, offers high clinical utility and helps save time and costs.^11,36^ Nevertheless, no universally accepted guidelines exist for genetic testing in pediatric NMDs. Although ES presents certain challenges, its implementation has expedited the diagnostic process for monogenic neuromuscular conditions. This study underscores the clinical value and management implications of ES. Although we support a genotype-first approach where NGS is accessible, we acknowledge that conventional investigations—such as electromyography/nerve conduction studies (EMG/NCS) and muscle biopsy—continue to play an important role. These modalities help refine phenotypes, localize pathology, and guide test selection, particularly in settings where NGS is not readily available or in cases with non-genetic or atypical presentations. Furthermore, they can assist in the interpretation of variants of uncertain significance. As costs continue to decrease, we recommend incorporating ES into the diagnostic workflow for children suspected of having genetic neuromuscular diseases. However, further research is necessary to evaluate the cost-effectiveness of ES in our setting. In the future, broader genomic approaches such as short-read and long-read genome sequencing may offer a more comprehensive alternative by detecting structural variants, deep intronic mutations, and other variant types not captured by current NGS platforms.

This study has some limitations. It was conducted at a single tertiary referral center, which may introduce a selection bias toward more complex or atypical presentations. Moreover, although our findings highlight the clinical utility of NGS, its cost-effectiveness requires further assessment, particularly in resource-limited environments. Additionally, the categorization of genetic testing results into positive, probable, possible, and negative by a multidisciplinary team—though guided by ACMG standards—involved some degree of subjectivity, particularly in the probable and possible categories. A consensus approach was employed to minimize bias, but potential subjectivities are acknowledged as a limitation of the study.

## Conclusions

This study demonstrates the substantial diagnostic yield and clinical utility of NGS in patients with suspected genetic NMDs who remain undiagnosed after conventional investigations. NGS testing not only provides definitive diagnoses but also informs personalized management strategies, including targeted therapies, medication optimization, and avoidance of unnecessary interventions. Although further research is needed to evaluate the cost-effectiveness of NGS, our findings support the integration of this technology into the diagnostic workup of NMDs.

## Supplementary information

Table S1

Table S2

Table S3

## Data Availability

The data supporting the findings of this study are available within the article and its supplementary material.
