# Supplementary material for: Next-generation sequencing for pediatric-onset neuromuscular disorders unresolved by conventional diagnostic methods
Source: Pediatr Res. 2025 Jun 10;98(6):2195–202. doi: 10.1038/s41390-025-04160-4 (PMC12811135; doi:10.1038/s41390-025-04160-4)
Supplement: Supplementary file 1 — Table S1 [file 41390_2025_4160_MOESM1_ESM.pdf]

Table S1. Single-gene and other genetic testing methods utilized in this cohort.

	**Test**	**Detected diseases**
Single-gene tests	*DMD*<br>- MLPA for *DMD*<br>- *DMD* gene sequencing	Duchenne muscular dystrophy
	SMN1<br>- PCR-RFLP for *SMN1*<br>- MLPA for *SMN1*	Spinal muscular atrophy
	PMP22 duplication/deletion: semiquantitative multiplex PCR, followed by DNA separation on a 5200 Fragment Analyzer System (Agilent)	Charcot–Marie–Tooth disease type 1A/ Hereditary neuropathy with liability to pressure palsies
	DMPK: triplet repeat primed PCR	Myotonic dystrophy type 1
	RYR1: Sanger sequencing of coding exons including 10, 11, 39-47 (NM_000540.3)	
	SCN4A: Sanger sequencing of 8 coding exons including 13, 14, and 19-24 (NM_000334.4)	Paramyotonia congenita
	CACNA1S and *SCN4A*: Sanger sequencing of coding regions<br>- *CACNA1S*: 5 common mutation in exon 11, 21, and 30<br>- *SCN4A*: 6 common mutation in exon 12 and 18	Hypokalemic periodic paralysis type 1<br>Hypokalemic periodic paralysis type 2
	Mitochondrial DNA<br>- Sanger sequencing: m.3243A>G (A3243G), m.8344A>G (A8344G), m.8993T>G (T8993G)<br>- Sanger sequencing: m.3460G>A (G3460A), m.11778G>A (G11778A), m.14484T>C (T14484C)<br>- gap-PCR: large deletions within the region from 3115 to 14873	Mitochondrial myopathies
	Targeted variant testing of known variant if positive family history	
Other genetic testing	Karyotype	Chromosomal abnormalities
	Methylation analysis and FISH	Prader–Willi syndrome
	FISH for 22q11 deletion	22q11 deletion syndrome

Abbreviations: FISH, fluorescent in-situ hybridization; gap-PCR, gap-polymerase chain reaction; MLPA, multiplex ligation-dependent probe amplification; PCR-RFLP, polymerase chain reaction-restriction fragment length polymorphism analysis
