# Supplementary material for: Next-generation sequencing for pediatric-onset neuromuscular disorders unresolved by conventional diagnostic methods
Source: Pediatr Res. 2025 Jun 10;98(6):2195–202. doi: 10.1038/s41390-025-04160-4 (PMC12811135; doi:10.1038/s41390-025-04160-4)
Supplement: Supplementary file 2 — Table S2 [file 41390_2025_4160_MOESM2_ESM.pdf]

Table S2. Clinical features and the impact of genetic diagnosis on clinical management.

Patient ID	Sex	AAO	Clinical features	Conventional investigation	Disease gene (phenotype /MIM number)	MOI	Impact on clinical management				
							Investigation	Treatment	Surveillance	Family counseling	Prognostication
**Group 1: Inherited myopathy**											
CM1	M	8Y6M	• Back pain and difficulty squatting<br>• Proximal muscle weakness<br>• Rigid spine, scoliosis<br>• Scapular winging	• CK 1931 U/L<br>• MLPA for DMD: negative<br>• Edx: irritable myopathy<br>• Muscle biopsy: myofibrillar myopathy	***FHL1***<br>(Myopathy, X-linked, with postural muscle atrophy/ 300696)	XR	-	-	Cardiac and respiratory involvement	• Family testing (father, mother)	Variable
CM2	F	27D	• Dilated cardiomyopathy<br>• Delay motor milestone<br>• Proximal muscle weakness	• Plasma amino acid: NL<br>• Urine organic acid: NL	***MYH7***<br>(Cardiomyopathy, dilated, 1S/ 613426)	AD	Avoid muscle biopsy	-	Hypertrophic and dilated cardiomyopathy	• Family testing (father, mother)	Yes
CM3*	F	4Y	• Distal weakness<br>• Scoliosis<br>• Mild restrictive lung disease	• CK 591 U/L<br>• Edx: myopathic change	***MYH7***<br>(Laing distal myopathy/ 160500)	AD	-	-	Hypertrophic and dilated cardiomyopathy	• Family testing (mother)	Yes
CM4*	M	4Y6M	• Distal weakness<br>• Scoliosis<br>• Moderate restrictive lung disease	• CK 608 U/L<br>• Muscle biopsy: myopathic change<br>• Edx: myopathic change	***MYH7***<br>(Laing distal myopathy/ 160500)	AD	-	-	Hypertrophic and dilated cardiomyopathy	• Family testing (mother)	Yes
CM5	M	1Y	• Delay motor milestone<br>• Proximal muscle weakness<br>• Myopathic face<br>• Scoliosis<br>• Chronic respiratory insufficiency	• CK 70 U/L<br>• Muscle biopsy: nemaline myopathy	***NEB***<br>(Nemaline myopathy 2/ 256030)	AR	-	-	Respiratory and bulbar involvement	• Family testing (father, mother)	Yes
CM6	M	3M	• Severe scoliosis<br>• Malignant hyperthermia<br>• Myopathic face<br>• Proximal muscle weakness	• CK 279 U/L<br>• Muscle biopsy: tubular aggregate myopathy<br>• Edx: myopathic change	***RYR1***<br>(Congenital myopathy 1B/ 255320)	AR	-	-	Respiratory involvement	• Family testing (father, mother)<br>• Malignant hyperthermia risk	Yes
CM7	M	3M	• Delay head up<br>• Ptosis with opthalmoplegia<br>• Proximal musle weakness	• CK 148 U/L<br>• Anti-AchR: positive<br>• Edx: NL, RNS: NL	***RYR1***<br>(Congenital myopathy 1B/ 255320)	AR	Avoid muscle biopsy	Off immunosuppressive drugs (previously treated as MG)	Respiratory involvement	• Family testing (father, mother)<br>• Malignant hyperthermia risk	Yes
CM8	M	birth	• Hypotonia<br>• Elongated face, high arch palate<br>• Ptosis with opthalmoplegia<br>• Undescended testes<br>• Oromotor dysfunction	• CK 58 U/L<br>• Anti-AchR: negative<br>• Muscle biopsy: myopathic change with type 1 fiber predominance<br>• Edx: myopathic, RNS: negative	***RYR1***<br>(Congenital myopathy 1B/ 255320)	AR	-	-	Respiratory involvement	• Family testing (father, mother)<br>• Malignant hyperthermia risk	Yes
CM9	M	birth	• Hypotonia<br>• Myopathic face<br>• Ptosis with opthalmoplegia<br>• Proximal musle weakness	• CK 116 U/L, lactate 1.5 mmol/L	***RYR1***<br>(Congenital myopathy 1B/ 255320)	AR	Avoid muscle biopsy	-	Respiratory involvement	• Malignant hyperthermia risk	Yes
CM10	M	8Y9M	• Walking difficulty<br>• Proximal muscle weakness	• CK 95 U/L<br>• Muscle biopsy: non specific myopathic change	***RYR1***<br>(Congenital myopathy 1B/ 255320)	AD/AR	-	-	Respiratory involvement	• Family testing (mother)<br>• Malignant hyperthermia risk	Yes
CM11	F	12Y9M	• Severe scoliosis<br>• Facial weakness, high arch palate<br>• Proximal muscle weakness<br>• Rigid spine<br>• Moderate restrictive lung disease	• CK 168 U/L<br>• Edx: myopathic change	***SELENON***<br>(Congenital myopathy 3 with rigid spine/ 602771)	AR	Avoid muscle biopsy	-	Cardiac and respiratory involvement	-	Yes

CM12	M	birth	• Hypotonia, respiratory failure<br>• Finger flexion contracture<br>• Myopathic face<br>• Proximal muscle weakness<br>• Rigid spine<br>• Scoliosis<br>• Moderate restrictive lung disease<br>• Abernethy malformation	• CK 86 U/L<br>• Muscle biopsy: non-specific change	***TTN***<br>(Limb-girdle muscular dystrophy/ 608807)	AR	-	-	Cardiac and respiratory involvement	• Family testing (father, mother)	Yes
CM13	M	birth	• Hypotonia, respiratory failure<br>• Myopathic face<br>• Proximal muscle weakness<br>• Oromotor dysfunction	• CK 57 U/L<br>• Muscle biopsy: myopathic change	***TTN***<br>(Limb-girdle muscular dystrophy/ 608807)	AR	-	-	Cardiac and respiratory involvement	• Family testing (father, mother)	Yes
CM14	F	birth	• Arthrogryposis multiplex congenita<br>• Proximal muscle weakness<br>• Oromotor dysfunction<br>• Severe scoliosis<br>• Dilated cardiomyopathy	• CK 37 U/L	***TTN***<br>(Congenital myopathy 5 with cardiomyopathy/ 611705)	AR	Avoid muscle biopsy	-	Cardiac and respiratory involvement	• Family testing (father, mother)	Yes
CM15	M	6M	• Delay motor milestone<br>• Facial weakness<br>• Hypotonia	• CK 243 U/L<br>• MLPA for *SMN1*: negative	19q13.33q13.41 deletion	-	-	-	-	-	-
DMD1	M	3Y6M	• Proximal muscle weakness (LE)<br>• Calf pseudohypertrophy<br>• Restrictive lung disease	• CK 18930 U/L<br>• MLPA for *DMD*: negative	***DMD***<br>(Duchenne muscular dystrophy/ 310200)	XR	Avoid muscle biopsy	• Steroid<br>• ACEI/ARB	• Dilated cardiomyoathy<br>• Respiratory involvement<br>• Scoliosis	-	Yes
DMD2	M	3Y	• Frequent falling<br>• Proximal muscle weakness (LE)<br>• Calf pseudohypertrophy	• CK 12704 U/L<br>• MLPA for DMD: negative	***DMD***<br>(Duchenne muscular dystrophy/ 310200)	XR	Avoid muscle biopsy	• Steroid<br>• ACEI/ARB	• Dilated cardiomyoathy<br>• Respiratory involvement<br>• Scoliosis	• Family testing (mother)<br>• Cardiac surveillance in DMD-carriers	Yes
DMD3	M	7Y	• Frequent falling<br>• Proximal muscle weakness (LE)<br>• Calf pseudohypertrophy	• CK 15097 U/L<br>• MLPA for DMD: negative<br>• Muscle biopsy: dystrophinopathy	***DMD***<br>(Duchenne muscular dystrophy/ 310200)	XR	-	• Steroid<br>• ACEI/ARB	• Dilated cardiomyoathy<br>• Respiratory involvement<br>• Scoliosis	-	Yes
DMD4	M	NA	• Down syndrome<br>• Unexplained transaminitis<br>• Severe intellectual disability<br>• Proximal muscle weakness (LE)<br>• Calf pseudohypertrophy	• CK >22000 U/L, AST, ALT<br>• MLPA for DMD: negative<br>• Muscle biopsy: dystrophinopathy	***DMD***<br>(Duchenne muscular dystrophy/ 310200)	XR	-	• Steroid<br>• ACEI/ARB	• Dilated cardiomyoathy<br>• Respiratory involvement<br>• Scoliosis	• Family testing (mother)<br>• Cardiac surveillance in DMD-carriers	Yes
DMD5	M	4Y	• Tiptoe walking<br>• Proximal muscle weakness (LE)<br>• Calf pseudohypertrophy	• CK 15645 U/L<br>• MLPA for DMD: negative	***DMD***<br>(Duchenne muscular dystrophy/ 310200)	XR	Avoid muscle biopsy	• Steroid<br>• ACEI/ARB	• Dilated cardiomyoathy<br>• Respiratory involvement<br>• Scoliosis	-	Yes
DMD6	M	3Y6M	• Walking difficulty<br>• Proximal muscle weakness (LE)<br>• Ankle contractures	• CK 9196 U/L<br>• MLPA for DMD: negative	***DMD***<br>(Duchenne muscular dystrophy/ 310200)	XR	Avoid muscle biopsy	• Steroid<br>• ACEI/ARB	• Dilated cardiomyoathy<br>• Respiratory involvement<br>• Scoliosis	-	Yes
DMD7	M	5Y	• Frequent falling<br>• Proximal muscle weakness (LE)<br>• Calf pseudohypertrophy	• CK 16571 U/L<br>• MLPA for DMD: negative	***DMD***<br>(Duchenne muscular dystrophy/ 310200)	XR	Avoid muscle biopsy	• Steroid<br>• ACEI/ARB	• Dilated cardiomyoathy<br>• Respiratory involvement<br>• Scoliosis	-	Yes
DMD8	M	1Y3M	• Hypomelanosis of Ito<br>• Macrocephaly<br>• Delayed walking<br>• Proximal muscle weakness (LE)	• CK 16836 U/L<br>• MLPA for DMD: negative<br>• Muscle biopsy: dystrophinopathy<br>• Brain MRI: NL	***DMD***<br>(Duchenne muscular dystrophy/ 310200)	XR	-	• Steroid<br>• ACEI/ARB	• Dilated cardiomyoathy<br>• Respiratory involvement<br>• Scoliosis	• Family testing (mother)<br>• Cardiac surveillance in DMD-carriers	Yes
DMD9	M	5Y	• Proximal muscle weakness (LE)<br>• Calf pseudohypertrophy<br>• Severe obstructive sleep apnea	• CK 12139 U/L<br>• MLPA for DMD: negative	***DMD***<br>(Duchenne muscular dystrophy/ 310200)	XR	Avoid muscle biopsy	• Steroid<br>• ACEI/ARB	• Dilated cardiomyoathy<br>• Respiratory involvement<br>• Scoliosis	• Family testing (mother)<br>• Cardiac surveillance in DMD-carriers	Yes

DMD10	M	4Y	• Frequent falling<br>• Proximal muscle weakness (LE)<br>• Calf pseudohypertrophy	• CK 7708 U/L<br>• MLPA for DMD: negative	***DMD***<br>(Duchenne muscular dystrophy/ 310200)	XR	Avoid muscle biopsy	• Steroid<br>• ACEI/ARB	• Dilated cardiomyoathy<br>• Respiratory involvement<br>• Scoliosis	-	Yes
DMD11	M	5Y	• Frequent falling<br>• Proximal muscle weakness (LE)<br>• Calf pseudohypertrophy<br>• Scoliosis	• CK 9874 U/L<br>• MLPA for DMD: negative<br>• Muscle biopsy: dystrophinopathy	***DMD***<br>(Duchenne muscular dystrophy/ 310200)	XR	-	• Steroid<br>• ACEI/ARB<br>• Candidate for Ataluren	• Dilated cardiomyoathy<br>• Respiratory involvement<br>• Scoliosis	-	Yes
DMD12	M	3Y	• Waddling gait<br>• Proximal muscle weakness (LE)<br>• Calf pseudohypertrophy	• CK 9089 U/L<br>• MLPA for DMD: negative	***DMD***<br>(Duchenne muscular dystrophy/ 310200)	XR	Avoid muscle biopsy	• Steroid<br>• ACEI/ARB<br>• Candidate for Ataluren	• Dilated cardiomyoathy<br>• Respiratory involvement<br>• Scoliosis	• Family testing (mother)<br>• Cardiac surveillance in DMD-carriers	Yes
DMD13	M	4Y	• Tiptoe walking<br>• Proximal muscle weakness (LE)<br>• Calf pseudohypertrophy<br>• Delay speech	• CK 7129 U/L<br>• MLPA for DMD: negative<br>• Muscle biopsy: dystrophinopathy	***DMD***<br>(Duchenne muscular dystrophy/ 310200)	XR	-	• Steroid<br>• ACEI/ARB	• Dilated cardiomyoathy<br>• Respiratory involvement<br>• Scoliosis	-	Yes
DMD14	M	2Y	• Tiptoe walking<br>• Proximal muscle weakness (LE)<br>• Calf pseudohypertrophy	• CK 13661 U/L<br>• MLPA for DMD: negative<br>• Muscle biopsy: dystrophinopathy	***DMD***<br>(Duchenne muscular dystrophy/ 310200)	XR	-	• Steroid<br>• ACEI/ARB	• Dilated cardiomyoathy<br>• Respiratory involvement<br>• Scoliosis	-	Yes
DMD15	M	4Y	• Tiptoe walking<br>• Proximal muscle weakness (LE)<br>• Calf pseudohypertrophy<br>• Ankle contractures	• CK 5848 U/L<br>• MLPA for DMD: negative<br>• Muscle biopsy: end stage muscle disease	***DMD***<br>(Duchenne muscular dystrophy/ 310200)	XR	-	• Steroid<br>• ACEI/ARB	• Dilated cardiomyoathy<br>• Respiratory involvement<br>• Scoliosis	• Family testing (mother)<br>• Cardiac surveillance in DMD-carriers	Yes
DMD16	M	1Y3M	• Frequent falling<br>• Proximal muscle weakness (LE)<br>• Calf pseudohypertrophy	• CK 13287 U/L<br>• MLPA for DMD: negative	***DMD***<br>(Duchenne muscular dystrophy/ 310200)	XR	Avoid muscle biopsy	• Steroid<br>• ACEI/ARB<br>• Candidate for Ataluren	• Dilated cardiomyoathy<br>• Respiratory involvement<br>• Scoliosis	-	Yes
DMD17	M	1Y3M	• Frequent falling<br>• Proximal muscle weakness (LE)<br>• Calf pseudohypertrophy<br>• GDD, ADHD, autism<br>• Epilepsy	• CK 10247 U/L<br>• MLPA for DMD: negative	***DMD***<br>(Duchenne muscular dystrophy/ 310200)	XR	Avoid muscle biopsy	• Steroid<br>• ACEI/ARB<br>• Candidate for Ataluren	• Dilated cardiomyoathy<br>• Respiratory involvement<br>• Scoliosis	-	Yes
MD1	M	1Y3M	• Delay walking<br>• Proximal muscle weakness<br>• GDD<br>• Scoliosis	• CK 1279 U/L<br>• MLPA for DMD: negative	***CHKB***<br>(Muscular dystrophy, congenital, megaconial type/ 602541)	AR	Avoid muscle biopsy	-	Dilated cardiomyopathy	-	Yes
MD2	F	1Y3M	• Delay walking<br>• Proximal muscle weakness<br>• Ichthyosis<br>• Dilated cardiomyopathy since 9Y	• CK 1545 U/L<br>• MLPA for DMD: negative	***CHKB***<br>(Muscular dystrophy, congenital, megaconial type/ 602541)	AR	Avoid muscle biopsy	-	Dilated cardiomyopathy	-	Yes
MD3	F	1Y	• Proximal muscle weakness<br>• Proximal joint contracture<br>• Distal joint hyperlaxity<br>• Keratosis pilaris, keloids	• CK 623 U/L<br>• PCR-RFLP for SMN1: negative<br>• Muscle biopsy: myopathic change, SSCD	***COL6A1***<br>(Ullrich congenital muscular dystrophy 1A/ 254090)	AD	-	-	Respiratory and cardiac involvement	• Family testing (father, mother)	Yes
MD4	M	4M	• Severe scoliosis<br>• Hip dislocation<br>• Hypotonia<br>• Proximal muscle weakness<br>• Distal joint laxity	• CK 300 U/L<br>• Muscle biopsy: myopathic change, SSCD	***COL6A1***<br>(Ullrich congenital muscular dystrophy 1A/ 254090)	AD	-	-	Respiratory and cardiac involvement	• Family testing (father, mother)	Yes

MD5	F	1Y	• Hip dislocation<br>• Proximal muscle weakness<br>• Hypertrophic scar<br>• Distal joint laxity	• CK 424 U/L<br>• PCR-RFLP for SMN1: negative<br>• Muscle biopsy: myopathic change, SSCD	***COL6A1***<br>(Ullrich congenital muscular dystrophy 1A/ 254090)	AD	-	-	Respiratory and cardiac involvement	-	Yes
MD6	M	4Y	• Walking difficulty<br>• Proximal muscle weakness<br>• Proximal joint contracture<br>• Bethlem sign<br>• Distal joint laxity<br>• Keratosis pilaris, keloid<br>• Epilepsy since 16Y	• CK 815 U/L<br>• Edx: myopathic pattern<br>• Muscle biopsy: myopathic change	***COL6A1***<br>(Bethlem myopathy 1A /158810)	AD	-	-	Respiratory and cardiac involvement	-	Yes
MD7	M	birth	• Hypotonia<br>• Proximal muscle weakness<br>• Proximal joint contracture<br>• Rigid spine<br>• Distal joint laxity<br>• Keratosis pilaris<br>• Scoliosis	• CK 170 U/L<br>• PCR-RFLP for SMN1: negative<br>• Muscle biopsy: myopathic change, complete negative COL6, absent staining on COL6/COL6 double stain	***COL6A2***<br>(Ullrich congenital muscular dystrophy 1B/ 620727)	AR	-	-	Respiratory and cardiac involvement	• Family testing (father, mother)	Yes
MD8	F	1Y6M	• Left torticollis<br>• Delay motor milestone<br>• Proximal muscle weakness<br>• Distal joint laxity<br>• Keratosis pilaris<br>• Scoliosis	• Neck U/S: very small size of left sternocleidomastoid with predominately tendon component<br>• CK 283 U/L	***COL6A2***<br>(Ullrich congenital muscular dystrophy 1B/ 620727)	AD	Avoid muscle biopsy	-	Respiratory and cardiac involvement	-	Yes
MD9	F	1Y	• Right sternocleidomastoid (3W) and left torticollis (1Y)<br>• Proximal muscle weakness<br>• Distal joint laxity<br>• Keratosis pilaris<br>• Scoliosis	• CK 563 U/L<br>• Edx: myopathic pattern<br>• Muscle biopsy: myopathic change, faint COL6 staining	***COL6A2***<br>(Ullrich congenital muscular dystrophy 1B/ 620727)	AD	-	-	Respiratory and cardiac involvement	-	Yes
MD10	F	birth	• Hip dislocation<br>• Proximal muscle weakness<br>• Proximal joint contracture<br>• Hypertrophic scar<br>• Keratosis pilaris<br>• Distal joint laxity	• CK 86 U/L<br>• Muscle biopsy: myopathic change, SSCD	***COL6A3***<br>(Ullrich congenital muscular dystrophy 1C/ 620728)	AD	-	-	Respiratory and cardiac involvement	-	Yes
MD11	F	4M	• Delay motor milestone<br>• Elongated face<br>• Facial weakness<br>• High arch palate<br>• Proximal muscle weakness	• CK 737 U/L<br>• MLPA for SMN1: negative<br>• Muscle biopsy: myopathic change, focally positive (faint, patchy, partial-deficiency) of merosin stainning<br>• Brain MRI: diffuse and bilateral symmetrical T2 hyperintense white matter lesion with spared corpus collosum and corticospinal tract	***LAMA2***<br>(Muscular dystrophy, congenital, merosin deficient/ 607855)	AR	Brain MRI	-	Respiratory and cardiac involvement	• Family testing (father, mother)	Yes
MD12	F	birth	• Hypotonia<br>• GDD<br>• Proximal muscle weakness<br>• Pectus excavatum	• CK 3326 U/L<br>• Edx: myopathic pattern<br>• Muscle biopsy: dystrophic change, faint merosin staining<br>• Brain MRI: diffuse and bilateral symmetrical T2 hyperintense white matter lesion with spared corpus collosum and corticospinal tract	***LAMA2***<br>(Muscular dystrophy, congenital, merosin deficient/ 607855)	AR	Brain MRI	-	Respiratory and cardiac involvement	• Family testing (father, mother)	Yes

MD13*	M	6M	• Delay motor milestone<br>• Hypotonia<br>• Myopathic face<br>• High arch palate<br>• Proximal muscle weakness<br>• Scoliosis<br>• Severe restrictive lung disease	• CK 443 U/L<br>• Edx: myopathic pattern<br>• Muscle biopsy: dystrophic change, absent merosin staining<br>• Brain MRI: diffuse and bilateral symmetrical T2 hyperintense white matter lesion with spared corpus collosum and corticospinal tract	***LAMA2***<br>(Muscular dystrophy, congenital, merosin deficient/ 607855)	AR	Brain MRI	-	Respiratory and cardiac involvement	-	Yes
MD14*	F	6M	• Delay motor milestone<br>• Hypotonia<br>• Myopathic face<br>• High arch palate<br>• Proximal muscle weakness<br>• Scoliosis<br>• Hip dislocation<br>• Severe restrictive lung disease	• CK 2593 U/L<br>• Edx: myopathic pattern	***LAMA2***<br>(Muscular dystrophy, congenital, merosin deficient/ 607855)	AR	Avoid muscle biopsy	-	Respiratory and cardiac involvement	-	Yes
MD15	M	3M	• Delayed motor milestone<br>• Hypotonia<br>• Proximal muscle weakness	• CK 1046 U/L<br>• MLPA for SMN1: negative	***LAMA2***<br>(Muscular dystrophy, congenital, merosin deficient/ 607855)	AR	Avoid muscle biopsy	-	Respiratory and cardiac involvement	-	Yes
MD16	M	5M	• Delayed motor milestone<br>• Hypotonia<br>• Proximal muscle weakness<br>• Focal epilepsy	• CK 1432 U/L<br>• Muscle biopsy: dystrophic change, absent merosin staining<br>• Brain MRI: diffuse and bilateral symmetrical T2 hyperintense white matter lesion with spared corpus collosum and corticospinal tract	***LAMA2***<br>(Muscular dystrophy, congenital, merosin deficient/ 607855)	AR	Brain MRI	-	Respiratory and cardiac involvement	-	Yes
MD17	M	4M	• Delayed motor milestone<br>• Hypotonia<br>• Proximal muscle weakness<br>• Scoliosis	• CK 6066 U/L<br>• MLPA for SMN1: negative	***LAMA2***<br>(Muscular dystrophy, congenital, merosin deficient/ 607855)	AR	Avoid muscle biopsy	-	Respiratory and cardiac involvement	• Family testing (father, mother)<br>• Preimplantation genetic testing	Yes
MD18*	F	3Y	• Frequent falling<br>• Proximal muscle weakness<br>• Early contracture<br>• Dilated cardiomyopathy<br>• Atrial fibrillation<br>• Sinus node dysfunction	• Lactate 1.3 mmol/L<br>• Edx: myopathic pattern<br>• Muscle biopsy: dystrophic change	***LMNA***<br>(Emery-Dreifuss muscular dystrophy 3/ 616516)	AR	-	-	Cardiac conduction defect, dilated or hypertrophic cardiomyopathy	• Family testing (mother)<br>• Cardiac surveillance in carriers	Yes
MD19*	F	3Y	• Frequent falling<br>• Proximal muscle weakness<br>• Early contracture<br>• Dilated cardiomyopathy<br>• Atrial fibrillation	• CK 629 U/L	***LMNA***<br>(Emery-Dreifuss muscular dystrophy 3/ 616516)	AR	Avoid muscle biopsy	-	Cardiac conduction defect, dilated or hypertrophic cardiomyopathy	• Family testing (mother)<br>• Cardiac surveillance in carriers	Yes
MD20	F	4Y	• Tiptoe walking<br>• Proximal muscle weakness<br>• Early contracture<br>• Rigid spine<br>• Scoliosis	• CK 194 U/L<br>• MLPA for SMN1: negative	***LMNA***<br>(Emery-Dreifuss muscular dystrophy 2/ 181350)	AD	Avoid muscle biopsy	-	Cardiac conduction defect, dilated or hypertrophic cardiomyopathy	• Family testing (father, mother)	Yes
MD21	F	7M	• Delayed motor milestone<br>• Proximal muscle weakness<br>• Early contracture<br>• Scoliosis<br>• Severe obstructive sleep apnea<br>• Atrial fibrillation	• CK 756 U/L<br>• PCR-RFLP for SMN1: negative<br>• Muscle biopsy: dystrophic change	***LMNA***<br>(Emery-Dreifuss muscular dystrophy 2/ 181350)	AD	-	-	Cardiac conduction defect, dilated or hypertrophic cardiomyopathy	• Family testing (father, mother)	Yes

MD22	F	2Y	• Tiptoe walking<br>• Proximal muscle weakness<br>• Lumbar lordosis	• CK 3266 U/L<br>• PCR-RFLP for SMN1: negative<br>• Muscle biopsy: dystrophic change	***LMNA***<br>(Emery-Dreifuss muscular dystrophy 2/ 181350)	AD	-	-	Cardiac conduction defect, dilated or hypertrophic cardiomyopathy	• Family testing (father, mother)	Yes
MM1	F	6Y4M	• Abnormal gait<br>• Proximal weakness<br>• Facial weakness<br>• Hypertrophic cardiomyopathy<br>• Hypoventilation	• CPK 1540 U/L<br>• Alpha-1,4 glucosidase: 0.3 umol/L/h<br>• Edx: irritable myopathy<br>• Muscle biopsy: glycogen accumulation	***GAA***<br>(Glycogen storage disease 2/ 232300)	AR	-	Candidate for enzyme replacement therapy (Myozyme)	• Cardiomyopathy<br>• Arrhythmia<br>• Diaphragmatic weakness	• Family testing (father, mother, brother)	Yes
MM2	F	birth	• Prematurity, GA 32^{+6} weeks<br>• Polyhydramnios<br>• Profound peripheral hypotonia<br>• Bilateral ptosis<br>• Diaphragmatic paralysis	• CPK 231 U/L<br>• Lactate 1.4 mmol/L, NH_3 32.2 umol/L<br>• *DMPK*: absence of CTG repeat expansion<br>• Muscle biopsy: polyglucosan bodies with a Maltese Cross birefingent pattern under polarized light	***GBE1***<br>(Glycogen storage disease 4/ 232500)	AR	-	Discontinuation of treatment, extubation	• Cardiomyopathy<br>• Arrhythmia<br>• Hepatic dysfunction<br>• Urinary tract dysfunction	• Family testing (father, mother)<br>• Preimplantation genetic testing	Yes
MC1	M	3Y	• Episodic muscle stiffness<br>• Myotonia with warm-up phenomenon<br>• Muscle hypertrophy	• Edx: myotonic discharge with transient CMAP increase on short exercise test	***CLCN1***<br>(myotonia congenita, recessive/ 255700)	AR	-	• Lamotrigine<br>• Carbamazepine<br>• Phenytoin<br>• Acetazolamide	-	• Avoid depolarizing muscle relaxants, adrenaline, beta-adrenergic agonists, and propranolol	-
MC2	M	11Y	• Episodic muscle stiffness<br>• Myotonia with warm-up phenomenon<br>• Muscle hypertrophy	-	***CLCN1***<br>(myotonia congenita, dominant/ 160800)	AD	-	• Lamotrigine<br>• Carbamazepine<br>• Phenytoin<br>• Acetazolamide	-	• Avoid depolarizing muscle relaxants, adrenaline, beta-adrenergic agonists, and propranolol	-
**Group 2: Inherited neuropathy**											
CMT1	F	birth	• Feet and legs deformities<br>• Distal muscle atrophy<br>• Bilaterak hip and knee flexion contractures<br>• Areflexia	• CPK 113 U/L<br>• Edx:neurogenic change in lower extremities<br>• Brain and spine MRI: NL	***DYNC1H1***<br>(CMT2O/ 614228)	AD	-	-	-	-	Yes
CMT2	F	2Y	• Delay walking<br>• Distal weakness (LE>UE)<br>• Distal paresthesia (LE)<br>• Scoliosis<br>• Wheelchair-dependent since 7Y	• Edx: diffuse demyelinating sensorimotor polyneuropathy<br>• *PMP22* dup: negative	***EGR2***<br>(CMT1D/ 607678)	AD	-	-	-	-	Variable
CMT3	M	1Y6M	• Delay walking<br>• Distal weakness (LE>UE)<br>• Distal paresthesia (LE)<br>• Pes cavus, hammer toe, claw hand	• CK 167 U/L<br>• Edx: diffuse demyelinating sensorimotor polyneuropathy<br>• *PMP22* dup: negative	***EGR2***<br>(CMT1D/ 607678)	AD	-	-	-	-	Variable
CMT4	M	6Y	• Abnormal gait<br>• Distal weakness (LE), hip extensors predominant<br>• No abnormal hair	• Edx: diffuse axonal sensorimotor polyneuropathy<br>• *PMP22* dup: negative	***GAN***<br>(Giant axonal neuropathy 1/ 256850)	AR	• Brain MRI<br>• Nerve biopsy	Candidate for precision medicine (scAAV9/JeT-GAN)	• Optic atrophy<br>• Ataxia, spasticity, CN dysfunction	• Family testing (father, mother)	-
CMT5	F	8Y10M	• Abnormal gait<br>• Distal weakness (LE)<br>• Rhomberg sign<br>• Pes cavus	• Edx: diffuse intermediate sensorimotor polyneuropathy<br>• CSF: albuminocytologic dissociation	***GDAP1***<br>(CMT2K/ 607831)	AD	-	Off steroid (previously treated as CIDP)	-	• Family testing (mother)	Yes

CMT6	F	9Y	• Foot deformity (equinovarus)<br>• Distal weakness (LE>UE)<br>• Distal paresthesia (LE)	• Edx: diffuse axonal sensorimotor polyneuropathy<br>• *PMP22* dup: negative	***GDAP1***<br>(CMT2K/ 607831)	AD	-	-	-	-	Yes
CMT7	F	8Y	• Frequent falling<br>• Distal weakness (LE>UE)<br>• Distal muscle atrophy (LE>UE)<br>• Pes cavus	• CK 99 U/L<br>• Edx: diffuse axonal sensorimotor polyneuropathy<br>• *PMP22* dup: negative	***GDAP1***<br>(CMT2K/ 607831)	AR	-	-	Vocal cord paresis	• Family testing (mother)	Yes
CMT8	F	6Y	• Frequent falling<br>• Distal weakness (LE>UE)<br>• Distal muscle atrophy (LE>UE)<br>• Pes cavus, hammer toe, foot valgus	• Edx: diffuse axonal sensorimotor polyneuropathy<br>• *PMP22* dup: negative	***GDAP1***<br>(CMT2K/ 607831)	AD/AR	-	-	-	• Family testing (father, sister)	Yes
CMT9	F	6Y	• Tiptoe walking<br>• Distal weakness (LE>UE)<br>• Distal paresthesia (LE)<br>• Equinovarus	• Edx: diffuse axonal sensorimotor polyneuropathy<br>• *PMP22* dup: negative	***GDAP1***<br>(CMT2K/ 607831)	AD/AR	-	-	-	• Family testing (father, sister)	Yes
CMT10	M	12Y	• Distal weakness (LE)<br>• Distal paresthesia (LE)<br>• Equinovarus	• Edx: diffuse intermediate sensorimotor polyneuropathy	***GJB1***<br>(CMTX1/ 302800)	XD	-	-	-	-	Yes
CMT11	F	8Y	• Distal weakness (LE)<br>• Distal paresthesia (LE)<br>• Equinovarus	• Edx: diffuse demyelinating sensorimotor polyneuropathy<br>• *PMP22* dup: negative	***HK1***<br>(CMT4G/ 605285)	AR	-	-	-	-	-
CMT12	M	1Y	• Never achieved walking<br>• Distal weakness (LE)<br>• Distal paresthesia (LE)<br>• Bilateral hip subluxation	• CK 150 U/L<br>• Brain and spine MRI: NL<br>• Muscle biopsy: neurogenic change<br>• PCR-RFLP for *SMN1*: negative<br>• Edx: diffuse axonal sensorimotor polyneuropathy with secondary demyelination	***IGHMBP2***<br>(CMT2S/ 616155)	AR	-	-	• Autonomic involvement	-	Yes
CMT13	F	5Y	• Chronic wound on the lateral foot<br>• Distal weakness (LE>UE)<br>• Distal paresthesia (LE>UE)<br>• Equinovarus, pes cavus<br>• Chronic osteomyelitis	• Whole spine MRI: diffuse mild small size of the entired spinal cord<br>• Edx: diffuse motor polyneuropathy, not perform sensory studies<br>• Nerve biopsy: axonal loss with endoneural fibrosis	***IGHMBP2***<br>(CMT2S/ 616155)	AR	-	-	• Autonomic involvement	-	Yes
CMT14	F	7M	• Foot drop<br>• Distal weakness (LE>UE)<br>• Distal paresthesia (LE>UE)<br>• Equinovarus<br>• Wheelchair-dependent since 6Y	• Muscle biopsy: neurogenic change<br>• Edx: diffuse axonal sensorimotor polyneuropathy<br>• *PMP22* dup: negative	***IGHMBP2***<br>(CMT2S/ 616155)	AR	-	-	• Autonomic involvement	• Family testing (father, mother)	Yes
CMT15	F	3Y	• Abnormal gait<br>• Distal weakness (LE>UE)<br>• Distal paresthesia (LE)	• Edx: diffuse axonal sensorimotor polyneuropathy<br>• *PMP22* dup: negative	***MFN2***<br>(CMT2A2A/ 609260)	AD	-	-	• Optic atrophy	• Family testing (father)	Yes
CMT16	F	3Y	• Distal weakness (LE>UE)<br>• Pes cavus	• Edx: diffuse axonal sensorimotor polyneuropathy<br>• *PMP22* dup: negative	***MFN2***<br>(CMT2A2A/ 609260)	AD	-	-	• Optic atrophy	-	Yes

CMT17	F	1Y	• Foot drop<br>• Distal weakness (LE>UE)<br>• Bilateral optic atrophy (13Y)<br>• Wheelchair-dependent since 18Y	• Edx: diffuse sensorimotor polyneuropathy (undetermined type)<br>• *PMP22* dup: negative<br>• Brain MRI: diffuse cerebellar atrophy, Atrophic change of bilateral optic nerves, optic chiasm and optic tracts.	***MFN2***<br>(CMT2A2A/ 609260)<br>(CMT2A2B/ 617087)	AR	-	-	• Optic atrophy	• Family testing (father, mother)	Yes
CMT18	M	1Y	• Delay walking, tiptoe walking<br>• Distal weakness (LE>UE)	• CK 95 U/L<br>• Muscle biopsy: chronic denervation with re-innervation, marked endoneural fibrosis with decreased myelinated fibers<br>• PCR-RFLP for *SMN1*: negative<br>• Edx: diffuse sensorimotor polyneuropathy (undetermined type)<br>• *PMP22* dup: negative	***MFN2***<br>(CMT2A2A/ 609260)	AD	-	-	• Optic atrophy	• Family testing (father, mother)	Yes
CMT19	M	1Y9M	• Frequent falling<br>• Distal weakness (LE>UE)<br>• Distal paresthesia (LE>UE)	• Edx: diffuse axonal sensorimotor polyneuropathy<br>• *PMP22* dup: negative	***MFN2***<br>(CMT2A2A/ 609260)	AD	-	-	• Optic atrophy	• Family testing (father, mother)	Yes
CMT20	M	2Y	• Unable to jump<br>• Distal weakness (LE>UE)<br>• Distal paresthesia (LE)	• CK 89 U/L<br>• Edx: diffuse axonal sensorimotor polyneuropathy<br>• *PMP22* dup: negative	***MFN2***<br>(CMT2A2A/ 609260)	AD	-	-	• Optic atrophy	• Family testing (father, mother)	Yes
CMT21	F	2Y	• Walking difficulty<br>• Distal weakness (LE, UE)<br>• Pes planus	• Edx: diffuse axonal sensorimotor polyneuropathy	***MFN2***<br>(CMT2A2A/ 609260)	AD	-	-	• Optic atrophy	-	Yes
CMT22	F	1Y3M	• Abnormal gait<br>• Distal weakness (LE, UE)<br>• Distal paresthesia (LE, UE)<br>• Pes planus, engrifted hand<br>• Rhomberg sign	• Edx: diffuse axonal sensorimotor polyneuropathy<br>• PMP22 dup: negative	***MFN2***<br>(CMT2A2A/ 609260)	AD	-	-	• Optic atrophy	• Family testing (father, mother)	Yes
CMT23	M	2Y	• Equinovarus, pes cavus, claw toes<br>• Distal weakness (LE)<br>• Distal muscle atrophy (LE)	• Edx: diffuse sensorimotor polyneuropathy (undetermined type)<br>• PMP22 dup: negative	***MFN2***<br>(CMT2A2A/ 609260)	AD	-	-	• Optic atrophy	• Family testing (father, mother)	Yes
CMT24	F	8Y	• Calf pain<br>• Distal weakness (LE, UE)<br>• Distal paresthesia (LE, UE)	• Edx: diffuse axonal sensorimotor polyneuropathy<br>• PMP22 dup: negative	***MFN2***<br>(CMT2A2A/ 609260)	AD	-	-	• Optic atrophy	-	Yes
CMT25	F	1Y3M	• Delay walking<br>• GDD with articulation disorder<br>• Distal weakness (LE, UE)<br>• Genu recurvatum	• CK 207 U/L<br>• PCR-RFLP for SMN1: negative<br>• Edx: diffuse demyelinating sensorimotor polyneuropathy<br>• PMP22 dup: negative	***MPZ***<br>(CMT1B/ 118200)	AD	-	-	• Hearing loss	• Family testing (father, mother)	Yes
CMT26	M	1Y	• Congenital severe scoliosis<br>• Delay sitting<br>• Distal weakness (LE>UE)<br>• Genu recurvatum<br>• Sleep breathing disorder on BiPAP	• MLPA for SMN1: negative<br>• Edx: diffuse demyelinating sensorimotor polyneuropathy<br>• PMP22 dup: negative	***MPZ***<br>(CMT1B/ 118200)	AD	-	-	• Hearing loss	• Family testing (mother)	Yes

CMT27	M	4Y	• Delay walking<br>• Distal weakness (LE>UE)<br>• Scoliosis	• Edx: diffuse demyelinating sensorimotor polyneuropathy<br>• PMP22 dup: negative	***MPZ***<br>(CMT1B/ 118200)	AD	-	-	• Hearing loss	• Family testing (father, mother)	Yes
CMT28	M	12Y	• Frequent falling<br>• Distal weakness (LE>UE)<br>• Bilateral SNHL	• Edx: diffuse demyelinating sensorimotor polyneuropathy<br>• PMP22 dup: negative	***NEFL***<br>(CMT1F/ 607734)	AD	-	-	• Hearing loss	• Family testing (father, mother)	-
CMT29	M	2Y	• Walking difficulty<br>• Distal weakness (LE, UE)<br>• Distal paresthesia (LE, UE)<br>• Pes cavus, hammer toe, claw hand<br>• Bilateral SNHL<br>• Neurogenic bladder (6Y)	• CK 271 U/L<br>• Edx: diffuse demyelinating sensorimotor polyneuropathy<br>• PMP22 dup: negative	***NEFL***<br>(CMT1F/ 607734)	AD	-	-	• Hearing loss	• Family testing (father, mother)	-
CMT30	M	1Y3M	• Walking difficulty<br>• Distal weakness (LE, UE)<br>• Distal paresthesia (LE)<br>• Pes cavus<br>• Left mild SNHL	• Edx: diffuse demyelinating sensorimotor polyneuropathy<br>• PMP22 dup: negative	***NEFL***<br>(CMT1F/ 607734)	AD	-	-	• Hearing loss	• Family testing (father, mother)	-
CMT31	M	3Y	• Distal weakness (LE)<br>• Distal paresthesia (LE)<br>• Rhomberg sign	• Edx: diffuse demyelinating sensorimotor polyneuropathy<br>• PMP22 dup: negative	***PMP2***<br>(CMT1G/ 618279)	AD	-	-	-	• Family testing (mother)	Yes
CMT32	M	1Y	• Delay walking and unsteady gait<br>• Distal weakness (LE, UE)<br>• Distal paresthesia (LE, UE)<br>• Ataxia	• Brain MRI: NL<br>• Edx: diffuse sensorimotor polyneuropathy (undetermined type)<br>• PMP22 dup: negative	***PMP22***<br>(CMT1E/ 118300)	AD	-	-	• Hearing loss	• Family testing (father, mother)	-
CMT33	M	1Y3M	• Delay walking<br>• Distal weakness (LE, UE)<br>• Distal paresthesia (LE, UE)	• Edx: diffuse sensorimotor polyneuropathy (undetermined type)<br>• PMP22 dup: negative	***PMP22***<br>(CMT1E/ 118300)	AD	-	-	• Hearing loss	-	-
CMT34	F	2Y	• Walking difficulty<br>• Distal weakness (LE, UE)<br>• Pes cavus	• Edx: diffuse demyelinating sensorimotor polyneuropathy	***SH3TC2***<br>(CMT4C/ 601596)	AR	-	-	• Hearing loss<br>• Scoliosis	-	Yes
HSN1	M	11Y	• Foot drop<br>• Distal weakness (LE>UE)<br>• Distal paresthesia (LE, UE)	• Edx: diffuse axonal sensorimotor polyneuropathy (sensory>motor)	***KIF1A***<br>(HSN2C/ 614213)	AR	-	-	-	-	-
HSN2	F	7M	• Tongue biting and self-multilation<br>• Constipation and bloody diarrhea<br>• Corneal ulcers (neuopathic keratitis)<br>• Severe Intellectual disability	• Edx: axonal predominate sensory polyneuropathy	***PRDM12***<br>(HSAN8/ 616488)	AR	-	-	-	• Family testing	-
**Group 3: Congenital myasthenic syndrome**											
CMS1	F	6M	• Bilateral ptosis<br>• Hypotonia<br>• Waddling gait	• CK 37 U/L<br>• Anti-AchR positive	***CHRNE***<br>(CMS4A/ 605809)	AD	Avoid muscle biopsy	• Discontinue AchEI (previously treated as ocular MG)<br>• Fluoxetine	-	• Family testing (father, mother)	Yes
CMS2	F	2W	• Bilateral ptosis with opthalmoplegia<br>• Recurrent respiratory failure	• CK 45 U/L, lactate 1.3 mmol/L<br>• Muscle biopsy: no specific change<br>• Edx: NL, RNS: decremental response of CMAP at 2-3 Hz stimulation	***COLQ***<br>(CMS5/ 603034)	AR	-	• Discontinue AchEI (previously treated as MG)<br>• Salbutamol, ephedrine	-	• Family testing (father, mother)	Yes

CMS3	F	birth	• Bilateral ptosis with opthalmoplegia<br>• Severe laryngomalacia<br>• Recurrent respiratory failure	• CK 22 U/L, lactate 1.2 mmol/L<br>• Anti-AchR negative<br>• Edx: NL, RNS: NL<br>• Muscle biopsy: non-specific change, no definite structural anomaly seen	***COLQ***<br>(CMS5/ 603034)	AR	-	• Avoid AchEI<br>• Salbutamol, ephedrine	-	• Family testing (father, mother)	Yes
CMS4	M	birth	• Bilateral ptosis with opthalmoplegia<br>• Marked hypotonia<br>• Severe scoliosis with excessive lumbar lordosis<br>• Recurrent respiratory failure	• CK 39 U/L<br>• Anti-AchR negative<br>• Edx: NL, RNS: decremental response of CMAP at 2-3 Hz stimulation<br>• Muscle biopsy: no specific change	***COLQ***<br>(CMS5/ 603034)	AR	-	• Avoid AchEI<br>• Salbutamol, ephedrine	-	-	Yes
CMS5	M	1Y	• GDD, FTT<br>• Hypotonia<br>• Recurrent pneumonia<br>• Bilateral ptosis with opthalmoplegia<br>• Gowers' sign<br>• Mild scoliosis with lordosis	• Plasma amino acid, urine organic acid: NL, NH_3 35 umol/L<br>• CK 96 U/L, lacate 2.4 mmol/L<br>• Brain MRI and MRS: NL<br>• Muscle biopsy: NL<br>• Edx: NL, RNS: decremental response of CMAP at 3 Hz stimulation	***COLQ***<br>(CMS5/ 603034)	AR	-	• Avoid AchEI<br>• Salbutamol, ephedrine	-	-	Yes
CMS6	M	10Y	• Proximal muscle weakness	• CK 159 U/L<br>• Edx: chronic myopathic change, RNS: decremental response of CMAP at 2 Hz stimulation<br>• Muscle biopsy: mild myopathic change, non-specific	***COLQ***<br>(CMS5/ 603034)	AR	-	• Avoid AchEI<br>• Salbutamol, ephedrine	• Respiratory involvement	-	Yes
CMS7	M	1M	• Bilateral ptosis with opthalmoplegia<br>• Proximal muscle weakness<br>• Scoliosis	• Edx: chronic myopathic change, RNS: decremental response of CMAP at 3 Hz stimulation with repetitive CMAP	***COLQ***<br>(CMS5/ 603034)	AR	Avoid muscle biopsy	• Avoid AchEI<br>• Salbutamol, ephedrine	• Respiratory involvement	-	Yes
CMS8*	M	19Y	• Bilateral ptosis<br>• Facial weakness<br>• Proximal muscle weakness	• Anti-AchR negative<br>• Muscle biopsy: mild myopathic change<br>• Edx: chronic myopathic change, RNS: decremental response of CMAP at 3 Hz stimulation	***RAPSN***<br>(CMS11/ 616326)	AR	-	• AchEI	• Respiratory involvement	• Family testing (sister)	Yes
CMS9*	F	5Y	• Bilateral ptosis<br>• Facial and bulbar weakness<br>• Proximal muscle weakness	• Anti-AchR negative<br>• RNS: decremental response of CMAP at 3 Hz stimulation	***RAPSN***<br>(CMS11/ 616326)	AR	-	• AchEI	• Respiratory involvement	• Family testing (brother)	Yes
CMS10	M	birth	• Congenital hypotonia<br>• Mild ptosis with ophthalmoplegia<br>• Facial and bulbar weakness<br>Proximak muscle weakness	• CK: NL<br>• Anti-AchR: negative<br>• RNS: decremental response of CMAP at 3 Hz stimulation	***RAPSN***<br>(CMS11/ 616326)	AR	Avoid muscle biopsy	• AchEI	• Respiratory involvement	• Family testing (father, mother)	Yes
**Group 4: Motor neuron disease**											
MND1	F	9M	• Hypotonia<br>• Myopathic face<br>• Proximal muscle weakness<br>• Areflexia<br>• Bilateral diaphragmatic paralysis<br>• Myoclonic epilepsy (develop after genetic diagnosis)	• CK 1475 U/L<br>• Muscle biopsy: neurogenic change<br>• Brain MRI: NL<br>• MLPA for *SMN1*: negative	***ASAH1***<br>(Spinal muscular atrophy with progressive myoclonic epilepsy/ 159950)	AR	-	• ASM<br>• Tracheostomy	• SNHL<br>• Epilepsy<br>• Scoliosis	• Family testing (father, mother, brother)<br>• Early diagnosis of her affected brother	Yes

Abbreviations: AAO, age at onset; ACEI, angiotensin-converting enzyme inhibitor; AchEI, acetylcholine esterase inhibitor; AD, autosomal dominant; ADHD, attention deficit hyperactive disorder; AMC, arthrogryposis multiplex congenita; AR, autosomal recessive; ARB, angiotensin receptor blocker; BiPAP, bilevel positive airway pressure; CIDP, chronic inflammatory demyelinating polyradiculoneuropathy; CK, creatine kinase (normal: < 190 U/L); CM, congenital myopathy; CMAP, compound muscle

action potential; CMS, congenital myasthenic syndrome; CN, cranial nerve; COL6, collagen 6; CV, conduction velocity; DMD, Duchenne muscular dystrophy; ECG, electrocardiogram; Edx, electrodiagnostic testing; EEG, electroencephalogram; EF, ejection fraction (from echocardiogram); F, female; GA, gestational age; GDD, global developmental delay; LE, lower extremities; LVNC, left ventricular noncompaction; M, male; MD, muscular dystrophy; MLPA, multiplex ligation-dependent probe amplification; MOI, mode of inheritance; MRI, magnetic resonance imaging; MRS, magnetic resonance spectroscopy; NIV, noninvasive ventilation; NL, normal; OSA, obstructive sleep apnea; PCR-RFLP, polymerase chain reaction-restriction fragment length polymorphism; PFT, pulmonary function test; RNS, repetitive nerve stimulation; SNHL, sensoineural hearing loss; SSCD, sarcolemma specific collagen deficiency; UE, upper extremities; XD, X-linked dominant; XR, X-linked recessive

* sibling relationship

In the patient-ID column, CM, DMD, MD, MM, MC, CMT, HSN, CMS, and MND represent patients clinically diagnosed with congenital myopathies, Duchenne muscular dystrophy, other muscular dystrophies, metabolic/mitochndrial myopathies, muscle channelopathy, Charcot–Marie–Tooth disease, hereditary sensory neuropathy, congenital myasthenic syndrome, and motor neuron disease, respectively.

In the AAO column, D, M, W, and Y represent day(s), month(s), week(s), and year(s), respectively.

In the conventional investigation column, Edx with CV of < 35 m/s in the upper extremity nerve indicated the demyelinating type, a CV of 35–45 m/s was classified as intermediate, and a CV of > 45 m/s was considered axonal.
