# Supplementary material for: Next-generation sequencing for pediatric-onset neuromuscular disorders unresolved by conventional diagnostic methods
Source: Pediatr Res. 2025 Jun 10;98(6):2195–202. doi: 10.1038/s41390-025-04160-4 (PMC12811135; doi:10.1038/s41390-025-04160-4)
Supplement: Supplementary file 3 — Table S3 [file 41390_2025_4160_MOESM3_ESM.pdf]

Table S3. Molecular findings from next-generation sequencing

Patient ID	NGS	Gene	Reference sequence	Nucleotide change	Protein change	Zygosity	Parental mutation status	MOI	Publication	ACMG classification [a]	AF in gnomAD (Total)	Result
**Group 1: Inherited myopathy**												
CM1	ES	*FHL1*	NM_001159702.3	c.377G>A	p.Cys126Tyr	Hemizygous	Maternal allele	XR	[PMID: 24928078]	Likely pathogenic	Variant not found	Positive
CM2	GP	*MYH7*	NM_000257.4	c.2711G>A	p.Arg904His	Heterozygous	De novo	AD	[PMID: 22464770]	Pathogenic	0.0000006195	Positive
CM3 [b]	ES	*MYH7*	NM_000257.4	c.4807G>C	p.Ala1603Pro	Heterozygous	Maternal allele	AD	[PMID: 27387980]	Likely pathogenic	Variant not found	Positive
CM4 [b]	ES	*MYH7*	NM_000257.4	c.4807G>C	p.Ala1603Pro	Heterozygous	Maternal allele	AD	[PMID: 27387980]	Likely pathogenic	Variant not found	Positive
CM5	GP	*NEB*	NM_001164508.2	c.133_146del	p.Ser45Thrfs*48	Heterozygous	Maternal allele	AR	-	Pathogenic	0.0000012410	Probable
CM6	ES	*RYR1*	NM_000540.3	c.487C>T	p.Arg163Cys	Compound heterozygous	Paternal allele	AR	[PMID: 12124989]	Pathogenic	0.0000012390	Positive
			NM_000540.3	c.15089G>A	p.Arg5030His		Maternal allele		[PMID: 36697461]	Likely pathogenic	0.0000266400	
CM7	GP	*RYR1*	NM_000540.3	c.9499C>T	p.Arg3167*	Heterozygous	Paternal allele	AR	[PMID: 28818389]	Pathogenic	0.0000043380	Probable
CM8	ES	*RYR1*	NM_000540.3	c.10347+1G>A	p.?	Compound heterozygous	Maternal allele	AR	[PMID: 32655342]	Pathogenic	0.0000080560	Positive
			NM_000540.3	c.11314C>T	p.Arg3772Trp		Paternal allele		[PMID: 37996280]	Pathogenic	0.0000055770	
CM9	GP	*RYR1*	NM_000540.3	c.11321C>A	p.Ala3774Glu	Homozygous	Not confirmed	AR	-	Likely pathogenic	Variant not found	Positive
CM10	GP	*RYR1*	NM_000540.3	c.13244_13264del	p.Ala4415_Asp4421del	Heterozygous	Maternal allele	AD/AR	[PMID: 26994242]	VUS	0.0001139000	Possible
CM11	ES	*SELENON*	NM_020451.3	c.1406G>A	p.Arg469Gln	Homozygous	Not confirmed	AR	[PMID: 30932294]	Pathogenic	0.0000123900	Positive
CM12	ES	*TTN*	NM_001267550.2	c.2254C>A	p.Arg752Ser	Compound heterozygous	Maternal allele	AR	[PMID: 36697461]	VUS	0.0000006200	Possible
		TTN	NM_001267550.2	c.38876-2A>C	p.?		Paternal allele		[PMID: 38937733, 36697461]	Pathogenic	0.0000056110	
CM13	ES	*TTN*	NM_001267550.2	c.39109G>T	p.Glu13037*	Compound heterozygous	Paternal allele	AR	[PMID: 36697461]	Pathogenic	0.0000012390	Positive
		TTN	NM_001267550.2	c.53348T>C	p.Leu17783Pro		Maternal allele		[PMID: 36697461]	Likely pathogenic	Variant not found	
CM14	GP	*TTN*	NM_001267550.2	c.46966+5G>C	p.?	Compound heterozygous	Paternal allele	AR	-	Likely pathogenic	Variant not found	Positive
		TTN	NM_001267550.2	c.86188A>T	p.Lys28730*		Maternal allele		-	Likely pathogenic	Variant not found	
CM15	ES	19q13.33q13.41 deletion (87 protein-encoding genes with possible NMD gene: *MYH14* gene)					-	-	-	VUS	-	Possible
DMD1	ES	*DMD*	NM_004006.3	c.515_516dup	p.Ile173Serfs*36	Hemizygous	Not confirmed	XR	[PMID: 36697461]	Pathogenic	Variant not found	Positive
DMD2	GP	*DMD*	NM_004006.3	c.3277-1G>C	p.?	Hemizygous	Maternal allele	XR	[PMID: 27122458]	Pathogenic	Variant not found	Positive
DMD3	ES	*DMD*	NM_004006.3	c.3630del	p.Glu1211Lysfs*4	Hemizygous	Not confirmed	XR	[PMID: 25007885, 16770791]	Pathogenic	Variant not found	Positive
DMD4	GP	*DMD*	NM_004006.3	c.3917dup	p.Asp1307Argfs*4	Hemizygous	Maternal allele	XR	-	Pathogenic	Variant not found	Positive
DMD5	ES	*DMD*	NM_004006.3	c.5697del	p.Lys1899Asnfs*2	Hemizygous	Not confirmed	XR	[PMID: 31727011]	Pathogenic	0.0000008270	Positive
DMD6	ES	*DMD*	NM_004006.3	c.5932del	p.Arg1978Valfs*5	Hemizygous	Not confirmed	XR	-	Pathogenic	Variant not found	Positive
DMD7	ES	*DMD*	NM_004006.3	c.6614+2T>C	p.?	Hemizygous	Not confirmed	XR	[PMID: 25007885]	Pathogenic	Variant not found	Positive
DMD8	ES	*DMD*	NM_004006.3	c.6986del	p.Lys2329Serfs*9	Hemizygous	Maternal allele	XR	[PMID: 26911353]	Pathogenic	0.0000000000	Positive
DMD9	ES	*DMD*	NM_004006.3	c.7197dup	p.Lys2400Glufs*10	Hemizygous	Maternal allele	XR	-	Likely pathogenic	Variant not found	Positive

DMD10	ES	*DMD*	NM_004006.3	c.8086del	p.Leu2696Trpfs*30	Hemizygous	Not confirmed	XR	[PMID: 25007885, 36315559]	Pathogenic	Variant not found	Positive
DMD11	ES	*DMD*	NM_004006.3	c.9100C>T	p.Arg3034*	Hemizygous	Not confirmed	XR	[PMID: 32153624]	Pathogenic	0.0000016540	Positive
DMD12	ES	*DMD*	NM_004006.3	c.9484G>T	p.Glu3162*	Hemizygous	Maternal allele	XR	[PMID: 36697461]	Likely pathogenic	Variant not found	Positive
DMD13	ES	*DMD*	NM_004006.3	c.9649+5G>T	p.?	Hemizygous	Not confirmed	XR	[PMID: 20485447]	Likely pathogenic	Variant not found	Positive
DMD14	ES	*DMD*	NM_004006.3	c.10087_10098del	p.Thr3363_Gly3366del	Hemizygous	Not confirmed	XR	[PMID: 32962870]	Likely pathogenic	Variant not found	Positive
DMD15	ES	*DMD*	NM_004006.3	c.10097_10099del	p.Gly3366del	Hemizygous	Maternal allele	XR	[PMID: 19937601, 28859693]	Likely pathogenic	Variant not found	Positive
DMD16	ES	*DMD*	NM_004006.3	c.10108C>T	p.Arg3370*	Hemizygous	Not confirmed	XR	[PMID: 18583217]	Pathogenic	Variant not found	Positive
DMD17	ES	*DMD*	NM_004006.3	c.10108C>T	p.Arg3370*	Hemizygous	Not confirmed	XR	[PMID: 18583217]	Pathogenic	Variant not found	Positive
MD1	ES	*CHKB*	NM_005198.5	c.598del	p.Gln200Argfs*11	Homozygous	Not confirmed	AR	[PMID: 31926838]	Pathogenic	0.0000061960	Positive
MD2	GP	*CHKB*	NM_005198.5	c.598del	p.Gln200Argfs*11	Homozygous	Not confirmed	AR	[PMID: 31926838]	Pathogenic	0.0000061960	Positive
MD3	ES	*COL6A1*	NM_001848.3	c.850G>A	p.Gly284Arg	Heterozygous	De novo	AD	[PMID: 17785674]	Pathogenic	Variant not found	Positive
MD4	ES	*COL6A1*	NM_001848.3	c.868G>A	p.Gly290Arg	Heterozygous	De novo	AD	[PMID: 30706156]	Pathogenic	Variant not found	Positive
MD5	ES	*COL6A1*	NM_001848.3	c.887G>A	p.Gly296Glu	Heterozygous	Not confirmed	AD	-	Pathogenic	Variant not found	Probable
MD6	ES	*COL6A1*	NM_001848.3	c.1056+1G>C	p.?	Heterozygous	Not confirmed	AD	[PMID: 25749816]	Pathogenic	Variant not found	Probable
MD7	GP	*COL6A2*	NM_001849.4	c.580C>T	p.Gln194*	Homozygous	Paternal and maternal allele	AR	-	Pathogenic	Variant not found	Positive
MD8	ES	*COL6A2*	NM_001849.4	c.893G>A	p.Gly298Glu	Heterozygous	Not confirmed	AD	-	Pathogenic	Variant not found	Probable
MD9	GP	*COL6A2*	NM_001849.4	c.2995G>A	p.Glu999Lys	Heterozygous	Not confirmed	AD	-	VUS	0.0000920800	Possible
MD10	GP	*COL6A3*	NM_004369.4	c.6309G>A	p.Lys2103=	Heterozygous	Not confirmed	AD	[PMID: 34167565]	Pathogenic	Variant not found	Positive
MD11	ES	*LAMA2*	NM_000426.4	c.172T>C	p.Cys58Arg	Compound heterozygous	Paternal allele	AR	-	Likely pathogenic	0.0000006200	Positive
		LAMA2	NM_000426.4	c.5085dup	p.Ala1696Serfs*6		Maternal allele		[PMID:32904964]	Pathogenic	0.0000006200	
MD12	ES	*LAMA2*	NM_000426.4	c.283+1G>C	p.?	Heterozygous	Paternal allele	AR	-	Pathogenic	Variant not found	Probable
MD13 [b]	GP	*LAMA2*	NM_000426.4	c.2049_2050del	p.Arg683Serfs*21	Heterozygous	Not confirmed	AR	[PMID: 28804634]	Pathogenic	0.0001450000	Probable
		LAMA2	NM_000426.4	c.4714_4717del	p.Val1572Phefs*22	Heterozygous	Not confirmed		-	Likely pathogenic	0.0000006200	
MD14 [b]	GP	*LAMA2*	NM_000426.4	c.2049_2050del	p.Arg683Serfs*21	Heterozygous	Not confirmed	AR	[PMID: 28804634]	Pathogenic	0.0001450000	Probable
		LAMA2	NM_000426.4	c.4714_4717del	p.Val1572Phefs*22	Heterozygous	Not confirmed		-	Likely pathogenic	0.0000006200	
MD15	GP	*LAMA2*	NM_000426.4	c.2538-1G>C	p.?	Heterozygous	Not confirmed	AR	[PMID: 32028919]	Likely pathogenic	Variant not found	Possible
	ES	*LAMA2*	NM_000426.4	c.2718del	p.Phe906Leufs*169	Heterozygous	Not confirmed	AR	[PMID: 36697461]	Pathogenic	Variant not found	Probable
		LAMA2	NM_000426.4	c.7452-16T>G	p.?	Heterozygous	Not confirmed		[PMID: 36697461]	Likely pathogenic	Variant not found	
MD17	GP	*LAMA2*	NM_000426.4	c.5476C>T	p.Arg1826*	Compound heterozygous	Paternal allele	AR	[PMID: 32936536]	Pathogenic	0.0000589600	Positive
		LAMA2	NM_000426.4	c.9101_9104dup	p.His3035Glnfs*5		Maternal allele		[PMID: 32904964]	Pathogenic	0.0000049570	
MD18 [b]	GP	*LMNA*	NM_170707.4	c.364A>G	p.Lys122Glu	Homozygous	Maternal allele (heterozygous)	AR	-	Likely pathogenic	0.0000018590	Positive
MD19 [b]	GP	*LMNA*	NM_170707.4	c.364A>G	p.Lys122Glu	Homozygous	Maternal allele (heterozygous)	AR	-	Likely pathogenic	0.0000018590	Positive
MD20	ES	*LMNA*	NM_170707.4	c.1072G>A	p.Glu358Lys	Heterozygous	De novo	AD	[PMID: 34487530]	Pathogenic	Variant not found	Positive

MD21	ES	*LMNA*	NM_170707.4	c.1153_1155del	p.Glu385del	Heterozygous	De novo	AD	[PMID: 36697461]	Pathogenic	Variant not found	Positive
MD22	ES	*LMNA*	NM_170707.4	c.1381-2A>C	p.?	Heterozygous	De novo	AD	[PMID: 36697461]	Pathogenic	Variant not found	Positive
MM1	ES	*GAA*	NM_000152.5	c.1556T>C	p.Met519Thr	Compound heterozygous	Maternal allele	AR	[PMID: 33741225]	Pathogenic	0.0000030980	Positive
		GAA	NM_000152.5	c.1561G>A	p.Glu521Lys		Paternal allele		[PMID: 26830551]	Pathogenic	0.0000037180	
MM2	ES	*GBE1*	NM_000158.4	c.1496T>A	p.Met499Lys	Compound heterozygous	Maternal allele	AR	[PMID: 36697461]	Likely pathogenic	0.0000006210	Positive
		GBE1	NM_000158.4	c.1561A>T	p.Lys521*		Paternal allele		[PMID: 20058079, 36697461]	Pathogenic	0.0000006210	
MC1	GP	*CLCN1*	NM_000083.3	c.871G>A	p.Glu291Lys	Heterozygous	Not confirmed	AR	[PMID: 27415035]	Pathogenic	0.0000049570	Probable
		CLCN1	NM_000083.3	c.991G>T	p.Ala331Ser	Heterozygous	Not confirmed		[PMID: 34529042]	Likely pathogenic	0.0000037230	
MC2	ES	*CLCN1*	NM_000083.3	c.913G>A	p.Gly305Arg	Heterozygous	Not confirmed	AD	-	Likely pathogenic	Variant not found	Probable
**Group 2: Inherited neuropathy**												
CMT1	GP	*DYNC1H1*	NM_001376.5	c.1756T>C	p.Ser586Pro	Heterozygous	Assumed de novo (maternal confirmed)	AD	-	Likely pathogenic	Variant not found	Probable
CMT2	ES	*EGR2*	NM_000399.5	c.925C>T	p.Arg309Trp	Heterozygous	Not confirmed	AD	[PMID:16198564]	Pathogenic	Variant not found	Positive
CMT3	ES	*EGR2*	NM_000399.5	c.1231G>A	p.Asp411Asn	Heterozygous	Not confirmed	AD	[PMID: 27549087]	Likely pathogenic	Variant not found	Probable
CMT4	GP	*GAN*	NM_022041.4	c.27C>G	p.Asp9Glu	Compound heterozygous	Maternal allele	AR	-	VUS	0.0000006520	Possible
		GAN	NM_022041.4	c.1629C>A	p.Tyr543*		Paternal allele		-	VUS	Variant not found	
CMT5	ES	*GDAP1*	NM_018972.4	c.368A>G	p.His123Arg	Heterozygous	Assumed de novo (maternal confirmed)	AD	[PMID: 26525999]	Pathogenic	0.0000006200	Probable
CMT6	ES	*GDAP1*	NM_018972.4	c.368A>G	p.His123Arg	Heterozygous	Not confirmed	AD	[PMID: 26525999]	Pathogenic	0.0000006200	Probable
CMT7	GP	*GDAP1*	NM_018972.4	c.445G>T	p.Asp149Tyr	Compound heterozygous	Maternal allele	AR	[PMID: 15469949]	Pathogenic	0.0000006200	Positive
		GDAP1	NM_018972.4	c.482G>A	p.Arg161His		Not confirmed		[PMID:37058526, 12843336]	Pathogenic	0.0000024810	
CMT8	ES	*GDAP1*	NM_018972.4	c.533del	p.Asn178Thrfs*28	Heterozygous	Not confirmed	AD/AR	-	Likely pathogenic	Variant not found	Possible
CMT9	ES	*GDAP1*	NM_018972.4	c.767A>G	p.His256Arg	Heterozygous	Paternal allele	AD/AR	[PMID: 21692914]	Pathogenic	0.0000037170	Probable
CMT10	ES	*GJB1*	NM_000166.6	c.223C>T	p.Arg75Trp	Hemizygous	Not confirmed	XR	[PMID: 12460545]	Pathogenic	0.0000000000	Positive
CMT11	ES	*HK1*	NM_000188.3	c.1A>G	p.Met1?	Homozygous	Not confirmed	AR	-	Likely pathogenic	0.0000006270	Positive
CMT12	ES	*IGHMBP2*	NM_002180.3	c.547+1G>A	p.?	Heterozygous	Not confirmed	AR	[PMID: 28902413]	Pathogenic	0.0000093590	Possible
CMT13	ES	*IGHMBP2*	NM_002180.3	c.983_987del	p.Lys328Thrfs*46	Heterozygous	Not confirmed	AR	[PMID: 27450922]	Pathogenic	0.0000316000	Possible
CMT14	ES	*IGHMBP2*	NM_002180.3	c.2773del	p.His925Thrfs*53	Compound heterozygous	Maternal allele	AR	-	Pathogenic	Variant not found	Positive
		IGHMBP2	NM_002180.3	c.2362C>T	p.Arg788*		Paternal allele		[PMID: 29761130]	Pathogenic	0.0000247900	
CMT15	ES	*MFN2*	NM_014874.4	c.272T>G	p.Val91Gly	Heterozygous	Assumed de novo (paternal confirmed)	AD	-	Pathogenic	Variant not found	Probable
CMT16	ES	*MFN2*	NM_014874.4	c.280C>T	p.Arg94Trp	Heterozygous	Not confirmed	AD	[PMID: 19889647]	Pathogenic	Variant not found	Probable
CMT17	ES	*MFN2*	NM_014874.4	c.430G>A	p.Ala144Thr	Compound heterozygous	Maternal allele	AR	-	Likely pathogenic	Variant not found	Positive
		MFN2	NM_014874.4	c.707C>T	p.Thr236Met		Paternal allele		[PMID: 30373780]	Pathogenic	0.0000018590	

CMT18	ES	*MFN2*	NM_014874.4	c.472A>C	p.Lys158Gln	Heterozygous	Maternal allele	AD	-	VUS	Variant not found	Possible
CMT19	ES	*MFN2*	NM_014874.4	c.617C>T	p.Thr206Ile	Heterozygous	Maternal allele	AD	[PMID: 16437557]	Pathogenic	Variant not found	Positive
CMT20	ES	*MFN2*	NM_014874.4	c.817G>T	p.Val273Leu	Heterozygous	Paternal allele	AD	[PMID: 18316077, 16043786]	Likely pathogenic	Variant not found	Positive
CMT21	ES	*MFN2*	NM_014874.4	c.1071G>C	p.Lys357Asn	Heterozygous	Not confirmed	AD	[PMID: 15549395]	pathogenic	Variant not found	Probable
CMT22	ES	*MFN2*	NM_014874.4	c.1090C>T	p.Arg364Trp	Heterozygous	De novo	AD	[PMID: 28063088]	Pathogenic	Variant not found	Positive
CMT23	ES	*MFN2*	NM_014874.4	c.1090C>T	p.Arg364Trp	Heterozygous	De novo	AD	[PMID: 28063088]	pathogenic	Variant not found	Positive
CMT24	ES	*MFN2*	NM_014874.4	c.1091G>C	p.Arg364Pro	Heterozygous	Not confirmed	AD	[PMID: 20008656]	Pathogenic	Variant not found	Probable
CMT25	ES	*MPZ*	NM_000530.8	c.245A>G	p.Tyr82Cys	Heterozygous	De novo	AD	[PMID: 9633821]	Pathogenic	Variant not found	Positive
CMT26	GP	*MPZ*	NM_000530.8	c.292C>T	p.Arg98Cys	Heterozygous	Assumed de novo (maternal confirmed)	AD	[PMID: 30785048]	pathogenic	0.0000018590	Probable
CMT27	ES	*MPZ*	NM_000530.8	c.551del	p.Leu184Hisfs*68	Heterozygous	De novo	AD	-	Pathogenic	Variant not found	Positive
CMT28	ES	*NEFL*	NM_006158.5	c.280C>T	p.Leu94Phe	Heterozygous	De novo	AD	[PMID: 27862672]	Likely pathogenic	Variant not found	Positive
CMT29	ES	*NEFL*	NM_006158.5	c.280C>T	p.Leu94Phe	Heterozygous	De novo	AD	[PMID: 27862672]	Likely pathogenic	Variant not found	Positive
CMT30	ES	*NEFL*	NM_006158.5	c.293A>G	p.Asn98Ser	Heterozygous	De novo	AD	[PMID: 31393079]	Pathogenic	Variant not found	Positive
CMT31	ES	*PMP2*	NM_002677.5	c.155T>C	p.Ile52Thr	Heterozygous	Assume de novo (maternal confirmed)	AD	[PMID: 30249361]	Pathogenic	Variant not found	Probable
CMT32	ES	*PMP22*	NM_000304.4	c.215C>T	p.Ser72Leu	Heterozygous	De novo	AD	[PMID: 37296061]	Pathogenic	Variant not found	Positive
CMT33	ES	*PMP22*	NM_000304.4	c.251_253del	p.Phe84del	Heterozygous	Not confirmed	AD	[PMID: 11355152]	Pathogenic	Variant not found	Probable
CMT34	ES	*SH3TC2*	NM_024577.4	c.929dup	p.Ser312Valfs*18	Homozygous	Paternal and maternal allele	AR	-	Pathogenic	Variant not found	Positive
HSN1	GP	*KIF1A*	NM_001244008.2	c.2840del	p.Leu947Argfs*4	Homozygous	Paternal and maternal allele	AR	[PMID: 21820098]	Pathogenic	0.0000129000	Positive
HSN2	ES	*PRDM12*	NM_021619.3	c.570+2T>G	p.?	Compound heterozygous	Paternal allele	AR	-	pathogenic	0.0000020080	Positive
		PRDM12	NM_021619.3	c.796A>C	p.Thr266Pro		Maternal allele		-	Likely pathogenic	Variant not found	
**Group 3: Congenital myasthenic syndrome**												
CMS1	GP	*CHRNE*	NM_000080.4	c.865C>T	p.Leu289Phe	Heterozygous	De novo	AD	[PMID: 27779167]	pathogenic	Variant not found	Positive
CMS2	ES	*COLQ*	NM_005677.4	c.393+1G>A	p.?	Homozygous	Paternal and maternal allele	AR	[PMID: 34912755]	Pathogenic	0.0000006200	Positive
CMS3	ES	*COLQ*	NM_005677.4	c.393+1G>A	p.?	Homozygous	Paternal and maternal allele	AR	[PMID: 34912755]	Pathogenic	0.0000006195	Positive
CMS4	ES	*COLQ*	NM_005677.4	c.393+1G>A	p.?	Homozygous	Not confirmed	AR	[PMID: 34912755]	Pathogenic	0.0000006195	Positive
CMS5	ES	*COLQ*	NM_005677.4	c.393+1G>A	p.?	Homozygous	Not confirmed	AR	[PMID: 34912755]	Pathogenic	0.0000006195	Positive
CMS6	GP	*COLQ*	NM_005677.4	c.393+1G>A	p.?	Heterozygous	Not confirmed	AR	[PMID: 34912755]	Pathogenic	0.0000006195	Probable
		COLQ	NM_005677.4	c.1281C>T	p.Cys427=	Heterozygous	Not confirmed		[PMID: 38003406]	Likely pathogenic	0.0000055880	
CMS7	GP	*COLQ*	NM_005677.4	deletion exons 14-15		Homozygous	Not confirmed	AR	[PMID: 34912755]	Pathogenic	-	Positive
CMS8 [b]	GP	*RAPSN*	NM_005055.5	c.-210A>G	p.?	Heterozygous	Not confirmed	AR	[PMID: 22326364]	Pathogenic	0.0000182700	Probable
		RAPSN	NM_005055.5	c.484G>A	p.Glu162Lys	Heterozygous	Not confirmed		[PMID: 28495245]	Pathogenic	0.0000080580	
CMS9 [b]	ES	*RAPSN*	NM_005055.5	c.484G>A	p.Glu162Lys	Heterozygous	Paternal allele	AR	[PMID: 28495245]	Pathogenic	0.0000080580	Probable

CMS10	ES	*RAPSN*	NM_005055.5	c.752_754del	p.Cys251del	Compound heterozygous	Paternal allele	AR	-	VUS	Variant not found	Possible
		RAPSN	NM_005055.5	c.844C>T	p.Arg282Cys		Maternal allele	AR	-	VUS	0.0000037270	
**Group 4: Motor neuron disease**												
MND1	ES	*ASAH1*	NM_177924.5	c.1A>T	p.Met1?	Compound heterozygous	Paternal allele	AR	-	Pathogenic	0.0000112600	Positive
		ASAH1	NM_177924.5	c.770T>C	p.Leu257Pro		Maternal allele		[PMID: 30029679]	Pathogenic	Variant not found	

Abbreviations: AD, autosomal dominant; AF, allele frequency; AR, autosomal recessive; ES, exome sequencing; gnomAD, genome aggregation database (v4.1.0); GP, gene panel; MOI, mode of inheritance; VUS, variant of uncertain significance; XR, X-linked recessive

[a] According to the American College of Medical Genetics and Genomics interpretation guidelines [PMID: 25741868]

[b] Sibling relationship

In the patient-ID column, CM, DMD, MD, MM, MC, CMT, HSN, CMS and MND represent patients clinically diagnosed with congenital myopathies, Duchenne muscular dystrophy, other muscular dystrophies, metabolic/mitochndrial myopathies, muscle channelopathy, Charcot–Marie–Tooth disease, hereditary sensory neuropathy, congenital myasthenic syndrome, and motor neuron disease, respectively.
